# Overcoming Cancer Persister Cells by Stabilizing the *ATF4* Promoter G‐quadruplex

**DOI:** 10.1002/advs.202401748

**Published:** 2024-07-12

**Authors:** Chengmei Xiao, Yipu Li, Yushuang Liu, Ruifang Dong, Xiaoyu He, Qing Lin, Xin Zang, Kaibo Wang, Yuanzheng Xia, Lingyi Kong

**Affiliations:** ^1^ State Key Laboratory of Natural Medicines and Jiangsu Key Laboratory of Bioactive Natural Product Research School of Traditional Chinese Pharmacy China Pharmaceutical University Nanjing 210009 China; ^2^ Shenzhen Research Institute of China Pharmaceutical University Shenzhen 518057 China

**Keywords:** ATF4, coptisine, glutamine‐restrictive therapy, G‐quadruplex

## Abstract

Persister cells (PS) selected for anticancer therapy have been recognized as a significant contributor to the development of treatment‐resistant malignancies. It is found that imposing glutamine restriction induces the generation of PS, which paradoxically bestows heightened resistance to glutamine restriction treatment by activating the integrated stress response and initiating the general control nonderepressible 2‐activating transcription factor 4‐alanine, serine, cysteine‐preferring transporter 2 (GCN2‐ATF4‐ASCT2) axis. Central to this phenomenon is the stress‐induced ATF4 translational reprogramming. Unfortunately, directly targeting ATF4 protein has proven to be a formidable challenge because of its flat surface. Nonetheless, a G‐quadruplex structure located within the promoter region of *ATF4* (*ATF4*‐G4) is uncovered and resolved, which functions as a transcriptional regulator and can be targeted by small molecules. The investigation identifies the natural compound coptisine (COP) as a potent binder that interacts with and stabilizes *ATF4*‐G4. For the first time, the high‐resolution structure of the COP‐*ATF4‐*G4 complex is determined. The formation of this stable complex disrupts the interaction between transcription factor AP‐2 alpha (TFAP2A) and *ATF4*‐G4, resulting in a substantial reduction in intracellular ATF4 levels and the eventual death of cancer cells. These seminal findings underscore the potential of targeting the *ATF4‐*G4 structure to yield significant therapeutic advantages within the realm of persister cancer cells induced by glutamine‐restricted therapy.

## Graphical abstract

Glutamine deprivation triggers metabolic adaptation, leading to the emergence of cancer persister cells that rely on stress‐induced ATF4 transcriptional reprogramming. For the first time, it is found that natural compound coptisine can stabilize the *ATF4*‐G4 and subsequently disrupt its interaction with transcription factor TFAP2A, ultimately restoring the effectiveness of glutamine‐restricted cancer therapies. The solution structures of coptisine‐*ATF4*‐G4 complexes are also determined.

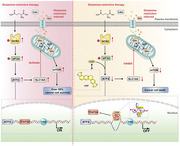

## 1 Introduction

Cancer cells enter a reversible persister state to evade death from chemotherapy or targeted agents.^[^ ^1^ ^]^ Persister cells (PS) are a type of cells that exhibit limited or infrequent division following cancer chemotherapy.^[^ ^2^ ^]^ These cells are distinguished by their slow proliferation rate, ability to adapt to their surrounding microenvironment, and capacity for phenotypic plasticity.^[^ ^3^ ^]^ PS is omnipresent in all types of clinical responses and can eventually and unpredictably give rise to metastatic relapses, known as invisible adversaries.^[^ ^3^ ^]^ Since the 1950s, glutamine (Q) has been widely identified as a crucial nutrient for cancer cells.^[^ ^4^ ^]^ Glutamine often observed as “depleted” in primary solid tumors.^[^ ^5^ ^]^ Glutamine depletion in response to the amino acid response pathway modulates DNA‐to‐RNA‐to‐protein cascades, coordinating genetic expression control to remodel metabolic reprogramming.^[^ ^6^ ^]^ Over ten distinct types of carcinomas have demonstrated sensitivity to glutamine deprivation, including lung cancer, pancreatic cancer, and breast cancer.^[^ ^7^ ^]^ Targeting the unique glutamine metabolic dependence of PS may reveal vulnerabilities that can be exploited therapeutically. Depriving cells of glutamine sensitizes prostate cancer cells to radiotherapy and kelch like ECH associated protein 1 (KEAP1)‐mutant lung adenocarcinoma.^[^ ^8^ ^]^ Furthermore, targeting glutamine metabolism at the transporter level^[^ ^9^ ^]^ and enzymes involved in glutamine synthesis and catabolism^[^ ^10^ ^]^ offers a promising approach for refining cancer medicine. Presently, 14 clinical trials targeting glutamine metabolism have been registered in ClinicalTrials (https://clinicaltrials.gov/). Notably, four ongoing clinical investigations have focused on non‐small‐cell lung cancer (NSCLC), specifically the CB‐839 trials (phase 1/2, NCT02071862, NCT02771626, NCT03965845, and NCT04265534).

Targeting the glutamine metabolic dependencies of PS is a viable approach. An example involves the potential of the timed inhibition of glutamine metabolism to eliminate persistent acute myeloid leukemia cells.^[^ ^11^ ^]^ This research underscores the potential of dietary interventions in treating various metabolic disorders, including cancer. Restricting dietary glutamine intake affects cerebellar glutamine levels and enhances survival rates in mouse brain tumor models.^[^ ^12^ ^]^ Nonetheless, a comprehensive understanding of the mechanisms by which persister cancer cells adapt to glutamine deprivation remains elusive, creating an urgent need for effective therapeutic strategies.

Glutaminase (GLS) has been implicated in glutamine addiction within tumors and exhibits oncogenic properties.^[^ ^13^ ^]^ Under glutamine restriction conditions, GLS transitions from a low‐activity dimer to a highly active polymer, thereby maximizing intracellular glutamine utilization at minimal concentrations. GLS filament formation is a physiological occurrence in solid tumors, suggesting the universality of glutamine depletion in solid tumors.^[^ ^14^ ^]^ The integrated stress response (ISR) is a complex signaling pathway observed in eukaryotic cells.^[^ ^15^ ^]^ Ryan et al. proposed an activating transcription factor 4 (ATF4)‐mediated ISR strategy to enhance metabolic adaptation during mitochondrial dysfunction.^[^ ^16^ ^]^ ATF4 plays a pivotal role in signaling cascades that promote both survival and apoptosis.^[^ ^17^ ^]^ Currently, multiple approaches are available for targeting ATF4 in cancer treatment.^[^ ^18^ ^]^ Initial strategies have focused on upstream eukaryotic translation initiation factor 2 (eIF2α) kinases, such as PKR‐like endoplasmic reticulum kinase (PERK), double‐stranded RNA‐dependent protein kinase (PKR), general control nonderepressible 2 (GCN2), or heme‐regulated eIF2α kinase (HRI), or on augmenting the activity of eIF2α phosphatase to inhibit eIF2α phosphorylation,^[^ ^19^ ^]^ thereby reducing overall ATF4 translation. Alternatively, inhibition of pathways regulated by ATF4 transcription offers an effective approach for targeting ATF4‐expressing cells.^[^ ^15b^ ^]^ ATF4 is a pivotal determinant of cellular fate during ISR activation, and stress‐induced translational reprogramming of ATF4 promotes PS survival. Consequently, inhibition of ATF4 has the potential to obstruct the persister phenotype in the context of nutritional restriction therapy.

Bioinformatics and experimental studies have revealed the accumulation of G‐quadruplex (G4)‐forming sequences in specific regions of the human genome, such as oncogene promoters, telomeres, and 5′‐UTR, influencing gene expression and genome stability.^[^ ^20^ ^]^ Small‐molecule drugs designed to stabilize G4 structures inhibit gene replication and transcription, thereby inducing cancer cell death.^[^ ^21^ ^]^ With the promising outcomes of G4‐targeting drugs in clinical trials,^[^ ^22^ ^]^ G4 structures have emerged as new therapeutic targets.^[^ ^23^ ^]^ The recent emphasis on ATF4 underscores its pivotal role in tumor progression.^[^ ^7f^ ^]^ The ATF4 protein is considered a poor and challenging target.^[^ ^18^ ^]^ Fortunately, we (and other researchers at the time of manuscript preparation) discovered that the *ATF4* promoter region can form a DNA G4 structure (*ATF4‐*G4).^[^ ^24^ ^]^ It is crucial to determine the function of this particular secondary structure and its role in pathological processes to ascertain its potential utility as a therapeutic target. Additionally, detailed structural information on *ATF4‐*G4 remains largely unknown, and the development of small molecules targeting *ATF4‐*G4 has not yet been reported. Natural products (NPs) are valuable sources of diverse structures that exhibit several biological attributes and are widely used in clinical diseases, especially cancer.^[^ ^25^ ^]^ Anti‐tumor alkaloids have demonstrated strong binding affinities for diverse DNA G4s, offering a novel strategy for anticancer drug development.^[^ ^26^ ^]^

In this study, we found that over 50% of persister cancer cells exhibit resilience to glutamine restriction therapy, leading to tolerance toward chemotherapeutic agents. We identified ATF4 as the key driver behind this persister phenotype. In the clinical cohort, 23% of cancer patients had high levels of ATF4 expression during glutamine depletion. Given the “undruggble” properity of ATF4 protein, we focused on the role of G4 structure within *ATF4* promoter region. We determined the utility of COP, serving as an *ATF4*‐G4 stabilizer, inhibiting *ATF4* expression both in *vivo* and in *vitro*. Using nuclear magnetic resonance (NMR), we determined the solution structure of *ATF4‐*G4 and its complex with coptisine (COP). Furthermore, we observed a unique metabolic feature of COP in glutamine restriction therapy, characterized by the limitation of glutamine transporter activity and the decrease of glutamine uptake that correlated with the severity of the disease. Moreover, we conducted a comprehensive screening and investigation of the transcriptional activity of the transcription factor AP‐2 alpha (TFAP2A) in relation to ATF4, both in the presence and absence of COP. Collectively, these findings shed light on tumor adaptive survival induced by glutamine deprivation.

## 2 Results

### 2.1 Glutamine‐Restrictive Therapy Fails to Enhance the Sensitivity of the Persister Cancer Cells

**Figure 1:**
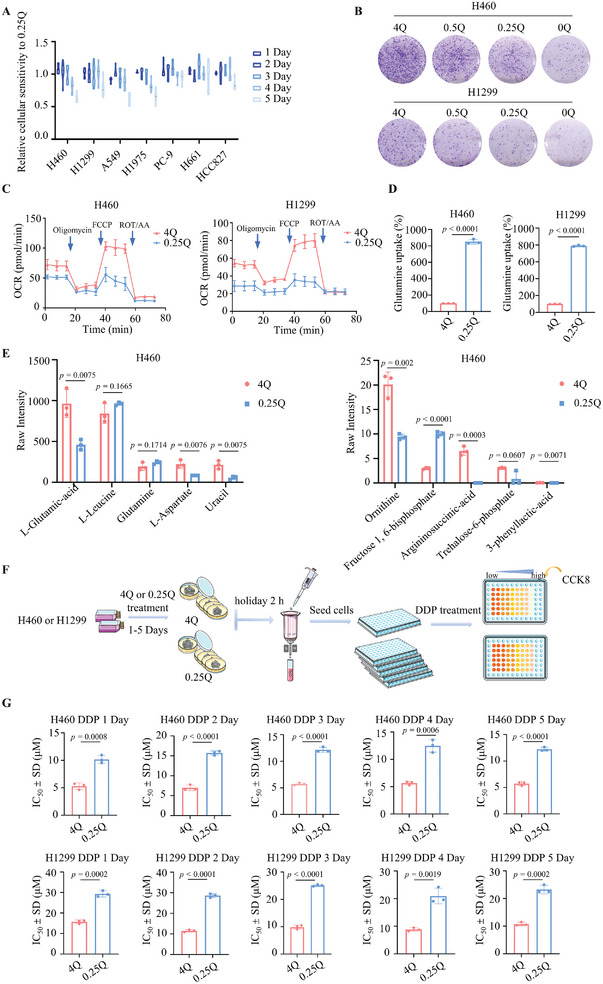
Glutamine‐restrictive therapy fails to enhance the sensitivity of the persister cancer cells. A) Sensitivity test to glutamine restriction in NCI‐H460, NCI‐H1299, NCI‐H1975, A549, PC‐9, NCI‐H661, and HCC827 cells. B) Clonogenic survival assay of NCI‐H460 and NCI‐H1299 cells under 4 mM to 0 mM glutamine conditions. Four thousand cells were cultured in six‐well plates for 10 days, followed by crystal violet staining and imaging. C) Seahorse XF assay measuring the OCR in NCI‐H460 and NCI‐H1299 cells under 4 mM or 0.25 mM glutamine conditions (*n* = 3 independent experiments). D) Glutamine uptake rate determination in NCI‐H460 and NCI‐H1299 cells cultured for 36 h in 4 mM or 0.25 mM glutamine using a glutamine assay kit (ab197011, Abcam) (*n* = 3 independent experiments). E) Analysis of differential metabolites between 4 mM and 0.25 mM glutamine conditions on H460 cells. The x‐axis represents groups, and the y‐axis represents expression (n = 3 independent experiments). F) CCK‐8 assay‐based analysis on the effect of glutamine (4 mM or 0.25 mM) on cisplatin treatment sensitivity in NCI‐H460 and NCI‐H1299 persister cells (*n* = 3 independent experiments). G) Generation of persister cells: NCI‐H460 and NCI‐H1299 cells treated with 4 mM or 0.25 mM glutamine for 1–5 days were switched to complete RPMI‐1640 medium for 2 h to create persister cells. Live cells were sorted, reseeded, and tested for cisplatin IC_50_. Then, live cells were harvested using the Dead Cell Removal kit (Miltenyi Biotec) (*n* = 3 independent experiments). Glutamine concentrations of 4 mM to 0 mM are represented by 4Q to 0Q, respectively. Data are shown as mean ± SD. *p < 0.05, **p < 0.01, ***p < 0.001, ****p < 0.0001. Data analyzed using two‐tailed Student's t‐tests (D, E, G) in GraphPad Prism 9.5.0.

We subjected seven distinct lung cancer cell lines to a glutamine‐deficient environment (0.25 mM glutamine) to assess their cell viability. Evidently, different cells exhibit distinct responses under similar conditions. Among these, NCI‐H460, A549, and NCI‐H1975 cells displayed heightened sensitivity to low‐glutamine conditions, whereas NCI‐H1299 cells demonstrated moderate sensitivity. In contrast, PC‐9, HCC827, and NCI‐H661 cells were insensitive to the glutamine‐restrictive milieu (**Figure** **1**A). Our research focuses on the effectiveness of glutamine deprivation therapies and overcoming the problem of cellular resistance that arises during the course of glutamine deprivation therapies. Therefore, the H460 and H1299 cell lines, which are highly and moderately sensitive to glutamine deprivation, respectively, were selected for subsequent experiments. The impacts of glutamine deficiency on cell survival and proliferation were concentration‐dependent, as depicted in Figure S1A–C (Supporting Information for survival) and Figure 1B along with Figure S1D (Supporting Information for proliferation). These outcomes underscore the unique metabolic reliance of cancer cells on glutamine to facilitate synthetic metabolism pathways.^[^ ^27^ ^]^ Glutamine, an amino acid conditionally essential, and abundant in both plasma and the intracellular amino acid pool, plays a pivotal role in bolstering the synthesis of glutathione—an ROS scavenger. Consequently, our findings demonstrated an increase in ROS levels due to the absence of glutamine (Figure S1E, Supporting Information). Glutamine is catabolized into α‐ketoglutaric acid, which subsequently enters the tricarboxylic acid (TCA) cycle to generate NADH and FADH2. Under conditions involving glutamine restriction, the presence of glutamine as a carbon and non‐essential amino acid nitrogen donor partially influences the dependence of cancer cells on other nutrients to fulfill the demands of the TCA cycle. This disruption of “glutamine addiction” impairs mitochondrial respiration, resulting in a reduced oxygen consumption rate (OCR), as depicted in Figure 1C. Furthermore, under low‐glutamine conditions, a significant increase in the uptake rate of glutamine by cancer cells was observed (Figure 1D). Energy metabolism analysis (Figure 1E) also confirmed that the intracellular glutamine content did not significantly differ between cells subjected to glutamine restriction and those treated with 4 mM glutamine, indicating a stress response aimed at maintaining the cancer cell state. Notably, the TCA cycle significantly reduced argininosuccinic acid, ornithine, L‐aspartate, L‐glutamic acid, and trehalose‐6‐phosphate levels. Likewise, the uracil content in the pentose phosphate pathway declined, whereas the levels of fructose 1,6‐bisphosphate in the glycolysis pathway increased.

Although glutamine restriction effectively promoted cancer cell death, notably the survival rate of these cells still exceeded 50% (Figure 1A). To further validate the anti‐cancer effects of glutamine constraint, we sorted PS of lung cancer to evaluate the tolerance to cisplatin under two different conditions involving 4 mM and 0.25 mM glutamine (Figure 1F). The results revealed that cells treated with 0.25 mM glutamine either exhibited heightened resistance to cisplatin (Figure 1G) or did not exhibit increased sensitivity (Figure S1F, Supporting Information). This led us to hypothesize that glutamine restriction induces a stress response, thereby triggering a protective adaptive mechanism.

### 2.2 Glutamine Deficiency Induces Integrated Stress Response to Activate the GCN2‐ATF4‐ASCT2 Axis

**Figure 2:**
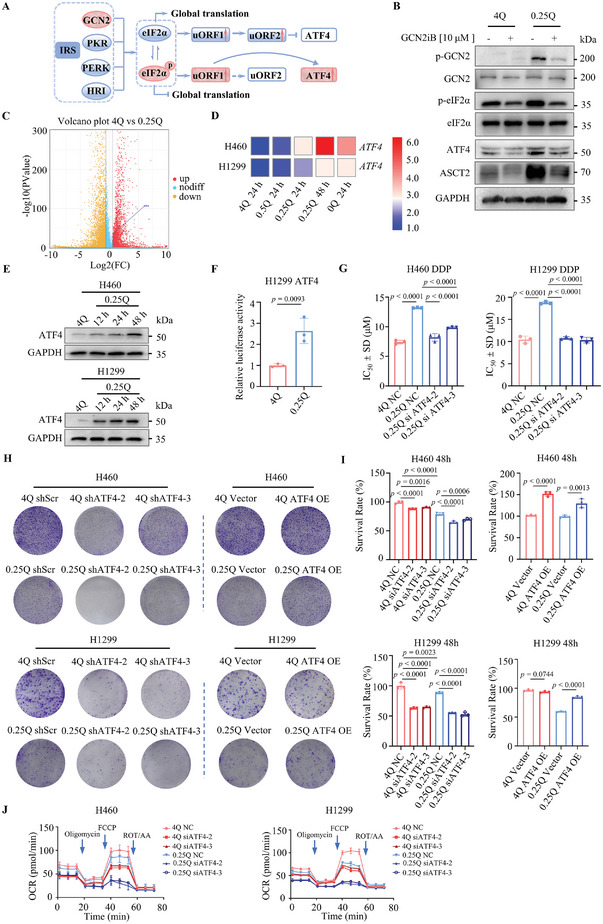
Glutamine deficiency induces integrated stress response to activate the GCN2‐ATF4‐ASCT2 axis. A) Schematic representation of GCN2‐eIF2α‐ATF4 axis activation in response to glutamine nutritional restriction. B) Immunoblotting analysis of indicated proteins in NCI‐H460 cells 48 h after treatment with GCN2iB, an ATP‐competitive inhibitor of the serine/threonine‐protein kinase general control nonderepressible 2 (GCN2). C) RNA‐Seq analysis compared differentially expressed genes in the H460 cell line (4Q versus 0.25Q). The ATF4 gene showed high expression in the 4Q versus 0.25Q fraction (*n* = 3 independent experiments). D) qRT‐PCR analysis of *ATF4* mRNA levels in NCI‐H460 and NCI‐1299 cells after 24 h of treatment with varying glutamine concentrations (4 mM, 0.5 mM, 0.25 mM, and 0 mM) and 48 h of treatment with 0.25 mM glutamine (*n* = 3 independent experiments, average of three technical replicates). The colors of the heatmap represent values of 2^−ΔΔCt^. E) Immunoblotting of ATF4 in NCI‐H460 and NCI‐H1299 cells after treatment with 0.25 mM glutamine for 12, 24, and 48 h. F) Luciferase activity of ATF4 in NCI‐H1299 cells treated with 4 mM or 0.25 mM glutamine for 24 h (*n* = 3 independent experiments). G) NCI‐H460 and NCI‐H1299 cells treated with 4 mM or 0.25 mM glutamine for 3 days were switched to complete RPMI‐1640 medium for 2 h to induce persister cells. These persister cells were then transfected with NC and siATF4, and the IC_50_ of cisplatin was assayed in live cell plates post‐transfection (*n* = 3 independent experiments). H) Clonogenic survival assay of NCI‐H460 and NCI‐H1299 cells transfected with shScramble, shATF4, Vector, and ATF4 OE plasmids under 4 mM or 0.25 mM glutamine. I) Survival rates of NCI‐H460 and NCI‐H1299 cells transfected with NC, siATF4, Vector, and ATF4 OE plasmids under 4 mM or 0.25 mM glutamine for 48 h. OE: Overexpression ATF4 (*n* = 3 independent experiments). J) Seahorse XF assay measuring OCR in NCI‐H460 and NCI‐H1299 cells after treatment with 4 mM or 0.25 mM glutamine following siATF4 transfection (*n* = 3 independent experiments). Immunoblots represent three similar results. Glutamine concentrations of 4 mM to 0 mM are represented by 4Q to 0Q, respectively. Data shown as mean ± SD. *p < 0.05, **p < 0.01, ***p < 0.001, ****p < 0.0001. Data analyzed using two‐tailed Student's t‐tests (F) and One‐way ANOVA (G, I) in GraphPad Prism 9.5.0.

The ISR pathway in eukaryotic cells is a multifaceted signaling network activated in response to a range of physiological alterations and diverse pathological conditions (**Figure** **2**A). When amino acids are depleted, the ISR, activated by GCN2, curtails the requirement for amino acids in protein synthesis, thereby mitigating stress levels.^[^ ^28^ ^]^ In glutamine deprivation conditions, our observations revealed that GCN2 (Figure 2B; Figure S2A,B, Supporting Information) triggered the phosphorylation of eIF2α at serine 51, which was distinct from that observed in HRI (Figure S2C, Supporting Information), PERK, and PKR. This eIF2α phosphorylation extends the ribosome scanning process at the open reading frame uORF1, located upstream of ATF4 transcript, consequently reinstating the transcriptional translation of ATF4^[^ ^18^, ^29^ ^]^ (Figure 2A). RNA‐Seq analysis demonstrated an increase in ATF4 expression when exposed to glutamine restriction (Figure 2C). The RNA (Figure 2D) and protein levels of ATF4 (Figure 2E; Figure S2D,E,G, Supporting Information), as well as the transcriptional activity of ATF4 (Figure 2F) increased in the highly and moderately glutamine‐deprivation‐susceptible NCI‐H460 and NCI‐H1299 cell lines under glutamine‐restricted conditions. Conversely, the expression levels of ATF4 in cell lines insensitive to glutamine restriction remained unaltered (Figure S2D,F, Supporting Information). Subsequent validation confirmed that the urgently induced ATF4 expression by ISR was suppressed by compound 968, a glutaminase inhibitor (Figure S2H, Supporting Information). This stress‐activated ATF4 acts as a chief transcription factor for genes responsive to stress, thus facilitating cellular recovery.^[^ ^28^ ^]^ Our hypothesis revolves around the potential of disrupting ATF4, the primary regulatory factor in ISR, to inhibit the adaptive survival reaction initiated by cancer cells via ISR. In PS induced with either 4 mM or 0.25 mM glutamine, the knockdown of ATF4 led to a reduction in cisplatin resistance linked to glutamine deprivation (Figure 2G). Our experiments demonstrated that the proliferation of NCI‐H460 and NCI‐H1299 cells under glutamine deficiency was significantly curtailed upon ATF4 knockdown (Figure 2H,I; Figure S3A–F, Supporting Information). Conversely, the introduction of exogenous ATF4 (Figure S2I, Supporting Information) undermined this inhibitory effect (Figure 2H,I; Figure S3A,F, Supporting Information). Furthermore, the knockdown of ATF4 disrupted mitochondrial respiration in NCI‐H460 and NCI‐H1299 cells (Figure 2J).

### 2.3 Glutamine‐Restrictive Therapy Benefits from the Low Levels of ATF4

**Figure 3:**
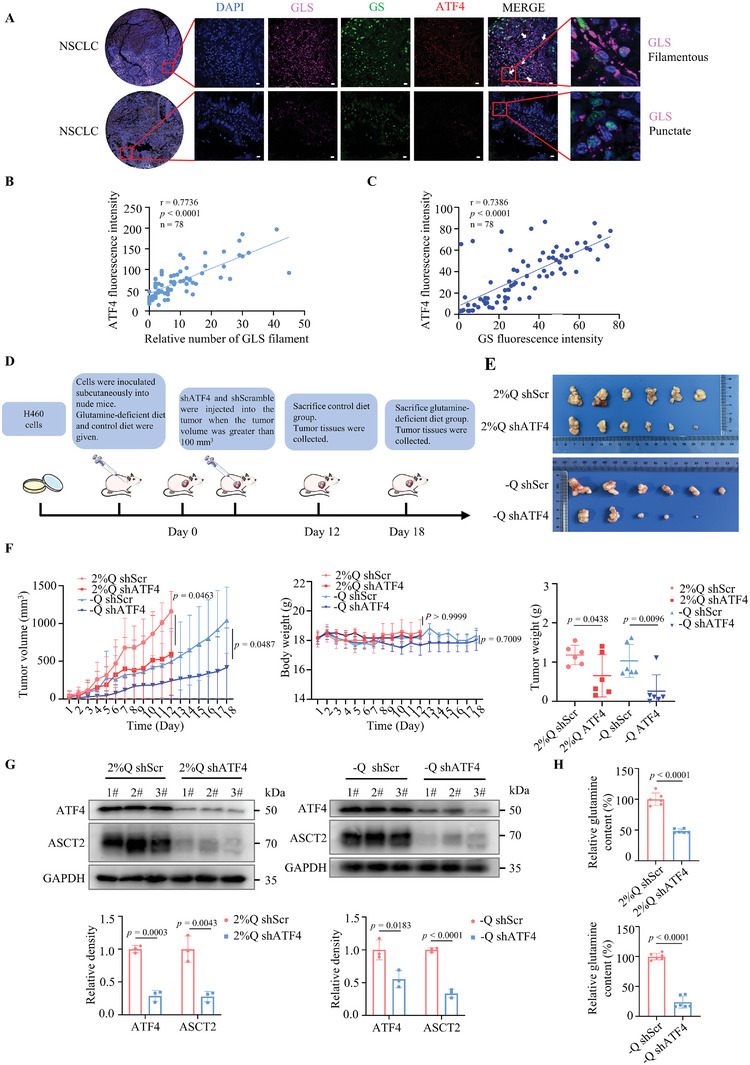
Glutamine‐restrictive therapy benefits from the low levels of ATF4. A) Tissue microarray of NSCLC patients with tissue immunofluorescence assay. Representative tissue immunofluorescence images with ATF4 (Red), GS (Green), and GLS (Pink). White arrows indicate GLS filaments. Scale bar: 10 µm. B) Correlation analysis between the number of relative GLS filament structures and positive cell density of ATF4 in the tissue microarray. C) Correlation analysis between positive cell density of GS and ATF4 in the tissue microarray. D) Schematic of tumors after intratumoral injection of shATF4 lentivirus with glutamine‐deficient diet and 2% glutamine diet in the established xenograft model (*n* = 6 per group). E) Tumor images (*n* = 6 per group). F) Tumor volume, body weight, and tumor weight of nude mice with intratumoral injection of shATF4 lentivirus and different glutamine diets (*n* = 6 per group). G) Protein expression levels in xenograft tumors after intratumoral injection of shATF4 lentivirus and different glutamine diets (*n* = 3 independent experiments). H) Relative glutamine content in different glutamine diet groups (*n* = 6 independent experiments). Glutamine concentrations of 2% and 0% glutamine diet are represented by 2%Q and ‐Q, respectively. Data are shown as mean ± SD. *p < 0.05, **p < 0.01, ***p < 0.001, ****p < 0.0001. Data were analyzed using two‐tailed Student's t‐tests (F, G, H) in GraphPad Prism 9.5.0. Source unprocessed blots are provided.

Glutamine is a critical nutrient for various solid tumors, particularly rapidly dividing tumor cells, which have an increased demand for glutamine. As a result, tumors growth is often associated with increased glutamine consumption, particularly in the central regions of solid tumors.^[^ ^30^ ^]^ As the tumor grows, elevated local glutamine utilization may reduce the glutamine supply to other areas, resulting in a heterogeneous distribution of glutamine throughout the tumor tissue.^[^ ^31^ ^]^ When cancer cells encounter a limited supply of glutamine, including regional glutamine deficiency or pharmacological blockade of glutamine metabolism, alternative intracellular adaptive mechanisms are employed to survive and continue to proliferate.^[^ ^31^, ^32^ ^]^ It is necessary to assess the potential mechanisms of tumor development under the inhibition of glutamine metabolism. Glutamine undergoes glutaminolysis by GLS prior to its contribution to bioenergetic processes and macromolecular synthesis.^[^ ^33^ ^]^ The induction of filament formation due to glutamine scarcity amplifies GLS activity and augments substrate‐binding affinity, thereby facilitating the efficient utilization of intracellular glutamine even at exceedingly low concentrations.^[^ ^14^ ^]^ Through the analysis of clinical tissue microarrays of NSCLC patients, we identified elevated expression of GLS in tumor tissues, accompanied by conspicuous GLS filament structures (**Figure** **3**A), suggesting a prevalent scarcity of glutamine in NSCLC.

Glutamine synthetase (GS), also known as GLUL, is a pivotal metabolic enzyme in cancer cells,^[^ ^34^ ^]^ facilitating the synthesis of glutamine from glutamate,^[^ ^35^ ^]^ which enables cells to persevere under glutamine‐depleted conditions, thereby influencing cancer circuitry and cellular outcomes.^[^ ^36^ ^]^ Our investigation revealed a positive association between GLS filament structure and ATF4 levels (Figure 3B). Similarly, a positive correlation was observed between the GS levels and ATF4 expression in human NSCLC tissues (Figure 3C). These insights garnered from tissue microarray analysis affirmed the link between glutamine depletion and heightened ATF4 levels in 23% of patients with NSCLC (18/78).

To assess the influence of glutamine restriction and ATF4 activity on NSCLC progression in vivo, we evaluated the growth of subcutaneous H460 xenograft tumors in mice fed a glutamine‐deficient diet (Figure 3D). This dietary regimen significantly impeded tumor growth, as evidenced by a reduction in tumor volume and endpoint tumor weight (Figure 3E,F). Glutamine restriction alone significantly inhibited tumor volume compared with the normal diet (Figure 3F). However, *ATF4* knockdown effectively inhibited tumor progression (Figure 3E,F). Immunohistochemistry (Figure S4A, Supporting Information) and western blotting (Figure 3G) analyses demonstrated that *ATF4* knockdown suppressed the expression of the glutamine transporter alanine, serine, and cysteine‐preferring transporter 2 (ASCT2), thereby affecting glutamine uptake and utilization in tumor cells during glutamine restriction (Figure 3H). *ATF4* knockdown curbed tumor cell proliferation in mice fed either a normal or glutamine‐deficient diet compared to that observed in the corresponding controls. Additionally, it reduced Ki67 levels, a widely used marker for assessing the proportion of proliferating cells in tumors.^[^ ^37^ ^]^ (Figure S4A, Supporting Information). Additionally, Kaplan‐Meier survival analysis indicated that lower ATF4 levels correlated with improved prognosis for patients with NSCLC (Figure S4B, Supporting Information). These findings suggest that dietary glutamine restriction activates the GCN2 signaling pathway and downstream ATF4 and ASCT2 expression, leading to an elevated glutamine uptake rate that sustains cancer cells. Inhibition of ATF4 counteracts the pro‐survival effect induced by stress and augments the responsiveness to glutamine‐based dietary therapy.

### 2.4 G‐Rich Tracts in the *ATF4* Proximal Promoter Region form a Specific G‐Quadruplex

**Figure 4:**
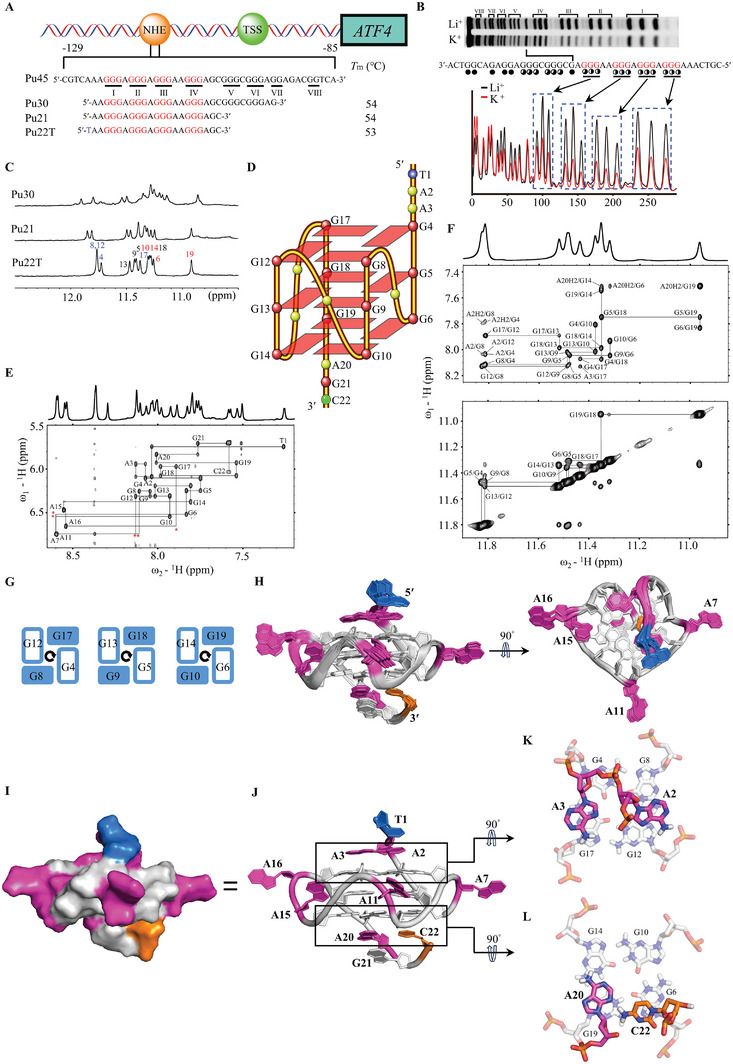
G‐rich tracts presented in *ATF4* promoter form a specific G‐quadruplex. A) Schematic of the human *ATF4* gene promoter and the G4‐forming region. The DNA sequence and its modifications are shown. The G‐runs with two or three continuous guanines are underlined and numbered. The guanines involved in the major G4 formation are colored in red and the mutations are colored in blue. The CD melting temperatures of 20 µM DNA in 5 mM K^+^‐containing solution are shown. B) DMS foot printing of the wild‐type Pu45 DNA showing guanines implicated in *ATF4‐*G4 formation. Pu45 DNA Sequence located in the *ATF4* proximal promoter region, spanning from ‐85 to ‐129 bp. In comparison to the darker blots observed in Li^+^‐containing solutions, the lighter protective blots evident in K^+^‐containing solutions undeniably signify the specific involvement of the 1st, 2nd, 3rd, and 4th G‐tracts in G‐tetrad formation, with a minor species possibly implicated in the 5th and 6th G‐tracts. C) 1D ^1^H‐NMR spectra of wild‐type and mutant *ATF4* gene promoter sequences. The G‐tetrad imino proton signals at the 5′‐end, middle, and 3′‐end are labeled in blue, black, and red respectively. Conditions: 150 µM DNA, pH 7, 50 mM K^+^. D) Folding topology of *ATF4‐*G4 with the Pu22T DNA sequence. E, F) H1′‐H6/H8 region, H1‐H1 and H1‐H8 regions from 2D‐NOESY spectra of Pu22T DNA in H_2_O with sequential assignment pathway. Missing connectivity is marked with red asterisks. G) Schematic diagram of the assigned three G‐tetrad planes of *ATF4‐*G4 by NMR experiments. H) Superposition of the 10 lowest energy NMR structures of *ATF4‐*G4. I) Surface view of a representative refined *ATF4‐*G4 structure. J) Cartoon representation of a refined *ATF4‐*G4 with partial numbering (PDB ID: 8WA4). Red, adenine; gray, guanine; blue, thymine; and orange, cytosine. K, L) 5′‐end and 3′‐end top views of *ATF4‐*G4.

These data provide compelling evidence that targeting the ATF4 signaling pathway is an appealing strategy for combination cancer therapy involving glutamine deprivation. Regrettably, ATF4 protein lacks a binding pocket for small molecules, which renders it challenging to target directly.^[^ ^18^ ^]^ Notably, targeting oncogene promoter G4s have been proven to be effective strategies in addressing the “undruggable” proteins, including MYC and KRAS.^[^ ^26^, ^38^ ^]^ This perspective prompted us to focus on *ATF4* gene transcriptional regulation by targeting the *ATF4* promoter G4s. The *ATF4* promoter sequence contained multiple G‐rich stretches, suggesting its potential to form various types of DNA G4 structures (**Figure** **4**A). To guide our investigation, we selected the Pu45 sequence, based on published G4‐specific ChIP‐seq data^[^ ^39^ ^]^ for further validation of the desired G4 structure formation using a dimethyl sulfate (DMS) footprinting assay, a method recognized for determining G4 formation under solution conditions. This assay is based on the fact that guanine N7 within a G‐tetrad involved in Hoogsteen hydrogen bonding remains protected from methylation and subsequent cleavage.^[^ ^40^ ^]^ The pivotal role of cations, particularly K^+^, in facilitating G4 formation is undisputed due to their superior coordination interactions with guanine O6s and lower dehydration energy. In contrast to the darker bands observed in Li^+^‐containing solutions, the lighter protective bands evident in K^+^‐containing solutions unequivocally indicate the involvement of the 1st, 2nd, 3rd, and 4th G‐tracts for the major *ATF4*‐G4 formation (Figure 4B). Furthermore, a minor G4 species may also be present, which is involved in the 5th and 6th G‐tracts (Figure 4B). We then employed the truncated wild‐type Pu21 sequence for further investigation, as it included all necessary G‐tracts for major *ATF4*‐G4 formation.

Through the 1D NMR ^1^H spectra (Figure 4C), we distinguished twelve distinct imino proton peaks in Pu21 positioned at chemical shifts of 10–12 ppm, which are characteristic of G4 structure formation. Elevated NMR baselines and miscellaneous peaks were observed, indicating the presence of minor structural species. Furthermore, the characteristic NMR peaks of the longer wild‐type Pu30 DNA containing the 5th and 6th G‐tracts were similar to those of the Pu21 sequence (Figure 4A,C). This further confirmed that major *ATF4*‐G4 was formed using the 1st, 2nd, 3rd, and 4th G‐tracts. Recognizing the importance of flanking residues in G4 formation and specific ligand recognition,^[^ ^41^ ^]^ we introduced thymine at the 5′‐end of Pu21, resulting in Pu22T. This variant exhibited twelve well‐defined imino proton resonances, indicating a stable three‐G‐tetrad‐stacked G4 structure suitable for subsequent structural determination (hereafter referred to as *ATF4*‐G4 for convenience). The consistency of the peak shape across different temperatures indicated the relative stability of *ATF4*‐G4 (Figure S5A, Supporting Information). Circular dichroism (CD) spectra displayed apparent parallel‐strand G4 characteristics in both Pu21 and Pu22T DNA, as evidently by the positive band at 264 nm and the negative band at 242 nm.^[^ ^42^ ^]^ Moreover, the native EMSA gel analysis unveiled the monomeric topology of free *ATF4*‐G4 (Figure S5D, Supporting Information). Additionally, the G to A mutation in each of the middle Gs within four consecutive G‐tracts leads to an *ATF4* G4‐mut sequence (Table S3, Supporting Information), which has been confirmed to be unable to form G4 structures through ^1^H‐NMR and CD experiments (Figure 4C; Figure S5B,C, Supporting Information).

For a more comprehensive analysis, we collected 2D NMR spectra, including ^1^H‐^1^H NOESY, ^1^H‐^1^H DQF‐COSY, and ^1^H‐^13^C HSQC, at different temperatures and mixing times in K^+^‐containing solutions (Figure 4E,F; Figure S5E, Supporting Information). Assignments were made for all resonances in the spectrum. The coherent core of G4 consisted of three G‐tetrads: G4‐G8‐G12‐G17, G5‐G9‐G13‐G18, and G6‐G10‐G14‐G19 (Figure 4G). Medium intensities of H1′‐H6/H8 NOE cross‐peaks and corresponding downfield C6/C8 chemical shifts indicated that all DNA residues adopted anti‐glycosidic torsion angles (Tables S5–S8 and Figure S5E, Supporting Information), in line with the characteristics of a parallel‐stranded G4.

To obtain a high‐resolution NMR solution structure of *ATF4*‐G4, we employed restrained molecular dynamics (MD) simulations based on the distance information derived from the NOESY spectra (Table 1; Table S6–S8, Supporting Information). Guided by a total of 522 NOE‐derived distances, 48 H‐bond restraints, and 22 torsion‐angle restraints, the resulting ten lowest energy structures displayed good convergence, with a heavy atom root‐mean‐square deviation (RMSD) of 0.56 ± 0.18 Å for the G‐tetrad core and 0.74 ± 0.24 Å for all residues (Table 1).

**Table 1:** NMR Restraints and Structural Statistics for the free *ATF4‐*G4 and its complex with coptisine.

	*ATF4‐*G4	Coptisine‐*ATF4‐*G4
NOE‐Based Distance Restraints		
Total	522	519
Intra‐residue	284	292
Inter‐residue		
Sequential	182	156
Long‐range	56	32
Ligand‐G4	–	39
Other Restraints		
Hydrogen bonds restraints	48	48
Torsion angles restraints	22	22
G‐tetrad planarity restraints	48	48
Structural Statistics		
Pairwise heavy atom RMSD [Å]		
G‐tetrad core	0.56 ± 0.18	0.53 ± 0.13
All residues	0.74 ± 0.24	0.59 ± 0.16
Restraint violations [Å]		
Max. NOE restraint violation	0.16	0.19
Mean NOE restraint violation	0.002 ± 0.012	0.002 ± 0.011

The high‐resolution solution unequivocally confirmed the well‐defined parallel‐stranded G4 structure (Figure 4H,I). At the 5′‐end site, three extended flanking residues formed a cohesive capping structure, with A2 and A3 stacking at the outer G‐tetrad (Figure 4J,K). This capping structure was supported by NOE cross‐peaks from A2H8 and A3H8 to the H1 of the 5′‐end tetrad‐forming Gs (Figure 4K; Table S6 and Figure S5F, Supporting Information). In contrast, the 3′‐end AGC residues adopted a distinct stacking arrangement from the 5′‐end segments (Figure 4J,L). The sequential residues G19, A20, and G21 formed a stacking structure, supported by the key NOE cross‐peaks of A20H8 to G19H8, A20H2 to G19H1, G14H1, G6H1, and A20H8 to G21H8 (Figure 4L; Table S7 and Figure S5G, Supporting Information). Two classic propeller loops, A7 and A11 (Figure 4H–J), conformed to the well‐documented arrangement.^[^ ^37^, ^39^, ^43^ ^]^ A unique feature of this structure was the double residue A15‐A16 loop, which has not been reported previously. Non‐parallel stacking primarily resulted from NOEs involving A15H8‐G17H8 and A15H8‐G18H8 (Figure 4H–J; Table S8 and Figure S5H, Supporting Information). In conclusion, *ATF4*‐G4 comprised a well‐defined three‐G‐tetrad stacked parallel G4 structure with a distinctive non‐parallel stacking 2nt‐loop and organized capping structures. CD experiments verified that the core mutant sequence does not possess G4 formation capability.

### 2.5 Coptisine Strongly Binds and Stabilizes the *ATF4‐*G4 to Hinder the Interaction Between TFAP2A and *ATF4* Promoter

**Figure 5:**
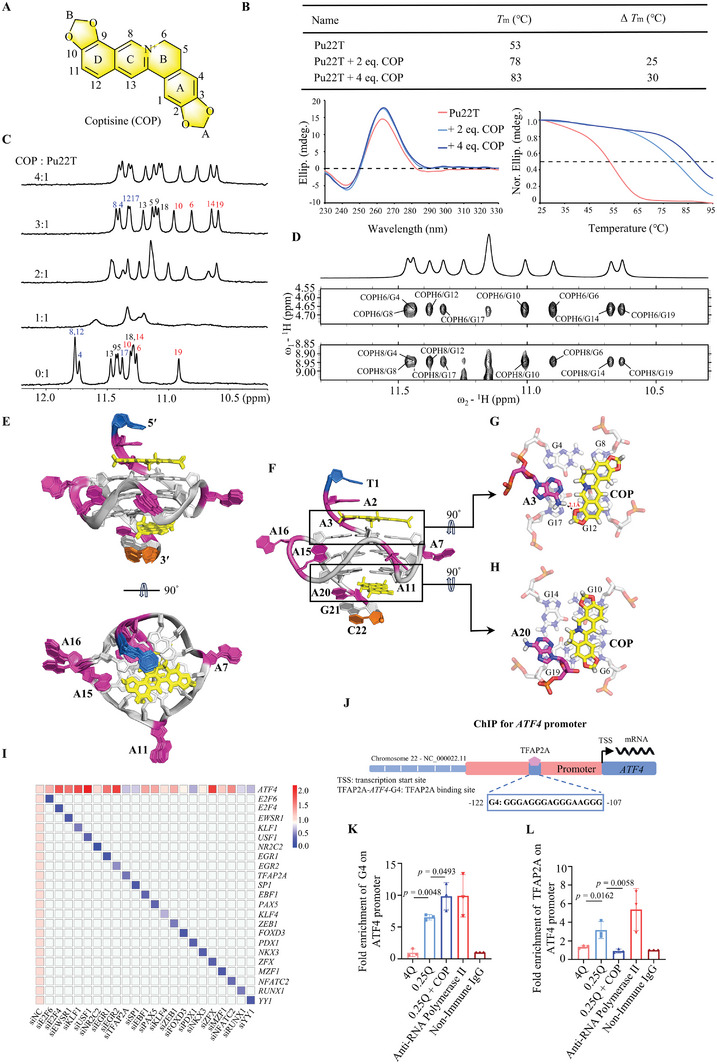
Coptisine strongly binds and stabilizes the *ATF4‐*G4 to hinder the interaction between TFAP2A and *ATF4* promoter. A) Chemical structure of Coptisine with numbering. (CAS NO. 6020‐18‐4) B) CD thermal melting curves and CD spectra of Pu22T DNA with coptisine. Conditions: 20 µM DNA, pH 7, 5 mM K^+^ solution. The ΔTm values of COP to the Pu22T DNA were determined. The melting temperature (Tm) was obtained at the intersection between the median of the fitted baselines and the melting curve. C) 1D ^1^H NMR titration of Pu22T DNA with COP. The G‐tetrad imino proton signals at the 5′‐end, middle, and 3′‐end are labeled in blue, black, and red respectively. Conditions: 150 µM DNA, pH 7, 50 mM K^+^ solution, 25 °C. D) 2D NMR spectra of *ATF4‐*G4 in complex with COP. Select regions of the 2D‐NOESY spectra of 2.4:1 COP‐*ATF4‐*G4 complexes in H_2_O showing intermolecular cross‐peaks between compound and DNA imino protons. Conditions: 2.11 mM Pu22T DNA, pH 7, 10 mM K^+^ solution, 25 °C. E) Superposition of the 10 lowest energy NMR structures of the COP‐*ATF4‐*G4. F) Cartoon representation of a refined COP‐*ATF4‐*G4 with partial numbering (PDB ID: 8Y2R). Red, adenine; gray, guanine; blue, thymine; orange, cytosine and yellow, COP. G, H) 5′‐end and 3′‐end top views of COP‐*ATF4‐*G4. I) Construct a small interfering library of 22 transcriptional regulatory ATF4 with the highest predicted score on Jasper and EPD websites, and screen out potential transcriptional factor TFAP2A (*n* = 3 independent experiments, average of three technical replicates). J) Schematic diagram of TFAP2A binding site in *ATF4* promoter region. K) Fold enrichment of G4 on *ATF4* promoter using ChIP‐qPCR analysis under 4Q or 0.25 mM glutamine conditions with or without COP treatment (20 µM) on NCI‐H1299 cells (12 h) (*n* = 3 independent experiments). L) Fold enrichment of TFAP2A on *ATF4* promoter using ChIP‐qPCR analysis under 4Q or 0.25 mM glutamine conditions with or without COP treatment (20 µM) on NCI‐H1299 cells (12 h) (*n* = 3 independent experiments). 4Q: 4 mM glutamine, 0.25Q: 0.25 mM glutamine, COP: coptisine chloride. The data are shown as mean values ± SD from triplicated samples. *p < 0.05, **p < 0.01, ***p < 0.001. Data were analyzed by One‐way ANOVA (K, L) in GraphPad Prism 9.5.0.

COP has demonstrated substantial potential as an anticancer drug in our recent study, which was attributed to its remarkable binding activity to parallel G4s^[^ ^38^ ^]^ (**Figure** **5**A). Given that *ATF4*‐G4 also adopts a parallel G4 structure, we investigated its binding to COP. Notably, we observed a significant enhancement in *ATF4*‐G4 thermal stability, with a notable 25 °C increase in a CD melting experiment (Figure 5B). To further assess the binding affinity, we determined their *K_d_* value to be ≈3.9 µM using a fluorescence‐based assay (Figure S6A, Supporting Information).

In a K^+^‐containing solution, we conducted ^1^H‐NMR titration experiments to probe the binding interactions between COP and *ATF4*‐G4 DNA. Free *ATF4*‐G4 exhibited 12 imino proton peaks, corresponding to three stacked G‐tetrads (Figure S5A, Supporting Information). Gradual addition of COP induced upfield shifts in nearly all imino proton resonances of free *ATF4*‐G4. At the lower drug ratio of 1:1, the imino proton peaks broadened, whereas at higher ratios of 2:1 and 3:1, the peaks sharpened, indicating the binding of one compound to each outer G‐tetrad via end‐stacking interactions (Figure 5C). Significantly, a new set of 12 distinct peaks emerged after COP addition, implying the formation of a dominant conformation within the COP‐*ATF4*‐G4 complexes.

The binding of COP to *ATF4*‐G4 was substantiated using NMR spectroscopy (Figure 5D; Figure S6B–D, Supporting Information), which generated well‐resolved NMR spectra suitable for high‐resolution structural analysis (Table S9, Supporting Information). A total of 519 NOE‐derived distance restraints were used to determine the COP‐*ATF4*‐G4 structure (Table 1). The final ten lowest energy structures demonstrated good convergence, with an RMSD of 0.59 ± 0.16 Å for all residues (Figure 5E and summarized in Table 1). COP adopted a 2:1 binding mode, optimizing its interaction with the outer G‐tetrads (Figure 5E–H). COP's positioning was supported by numerous NOE cross‐peaks, such as COPH6‐G4H1, COPH6‐G8H1, COPH6‐G12H1, COPH6‐G17H1, COPH8‐G4H1, COPH8‐G8H1, COPH8‐G12H1, COPH8‐G17H1, COPH6‐G6H1, COPH6‐G10H1, COPH6‐G14H1, COPH6‐G19H1, COPH8‐G6H1, COPH8‐G10H1, COPH8‐G14H1, and COPH8‐G19H1 (Figure 5D; Table S10, Supporting Information). Electrostatic interactions may arise between the positively charged COPN7 and the negatively polarized carbonyl groups of the tetrad guanine. Moreover, the resonances of COPHA‐G6H8, COPHB‐G17H8, COPHA‐G6H8, and COPHB‐G14H8 played a pivotal role in determining the stacking direction (Table S10, Supporting Information). Moreover, the higher affinity of COP for the 5′ end capping structure can be attributed to a plausible hydrogen bond between COP and A3H2 (Figure 5G; Figure S6E, Supporting Information). Considering their positions above the outermost tetrads, designing coptisine derivatives with longer alkyl chains could be a rational strategy to offer molecular guidance for enhancing the affinity and selectivity with drug‐like properties through hydrogen bonding and electrostatic interactions.

The overall binding mode of COP‐*ATF4*‐G4 closely resembles the reported COP‐*KRAS*‐G4 binding mode^[^ ^38^ ^]^ (Figure 5E–F). Each COP molecule recruited an adjacent flanking residue, forming a plane that was stacked over two external G‐tetrads (Tables S11 and S12, Supporting Information). The 2nt‐loop structure did not contribute to binding pocket formation, consistent with the fact that almost the same conformation was observed in both the *ATF4*‐G4 free structure and the COP‐*ATF4*‐G4 complex (Tables S8 and S13, Supporting Information). Moreover, clear assignments of intermolecular NOE cross‐peaks between the COP and A3 residues were observed at high threshold levels, suggesting multiple orientations of A3. For instance, intermolecular NOE cross peaks from COPH8 to A3H8 were observed, implying a potential flip of the A3 orientation by ≈180° from the determined conformations. However, limited NOE cross‐peaks hindered the precise structural determination of this minor species.

Numerous G4 sites and transcription factors that recognize G4 structures play pivotal roles in human chromatin regulation.^[^ ^44^ ^]^ Therefore, it is crucial to identify transcription factors that can bind to *ATF4*‐G4 to gain a comprehensive understanding of the biological functions of *ATF4*‐G4. Using JASPAR (http://jaspar.genereg.net/) and EPD (https://epd.epfl.ch//index.php), we identified twenty‐two transcription factors with predicted scores exceeding eight for potential regulation of *ATF4* gene expression (Figure 5I). A siRNA library was then constructed, and qRT‐PCR was used to validate the transcriptional effect of these factors on ATF4, resulting in the identification of the transcription factor TFAP2A (Figure 5J). Presently, in vitro analysis has suggested that TFAP2A binds to the palindrome motif GCCN3GGC, as well as some variants such as GCCN4GGC and GCCN3/4GGG.^[^ ^45^ ^]^ ChIP‐seq experiments have demonstrated that SCCYSRGGS (S = G or C, R = A or G, and Y = C or T) are the consensus sites for human TFAP2A.^[^ ^46^ ^]^ Moreover, TFAP2A has also been shown to bind to G4‐forming sequences.^[^ ^47^ ^]^ Therefore, we hypothesized that TFAP2A could potentially bind to the *ATF4*‐G4‐forming sequence. To validate our hypothesis, primer sequences were specifically designed for the *ATF4*‐G4‐forming region, and chromatin immunoprecipitation followed by quantitative polymerase chain reaction (ChIP‐qPCR) experiments were conducted. Firstly, ChIP‐qPCR was performed using anti‐G4 antibodies to confirm the presence of the G4 structure within the *ATF4* promoter region in the cells (Figure 5K). Subsequently, ChIP‐qPCR was conducted with TFAP2A antibodies to investigate TFAP2A binding to *ATF4‐*G4. The results showed that the DNA fragment for the *ATF4‐*G4 forming region was enriched by both the G4 antibody and TFAP2A antibody under 0.25 mM glutamine conditions, and COP stabilized the *ATF4‐*G4 and inhibited its interaction with TFAP2A (Figure 5K,L). These findings indicate that the binding of TFAP2A to the *ATF4‐*G4‐forming sequence is involved in the *ATF4* transcription regulation and small molecules can disrupt their interaction by stabilizing the *ATF4‐*G4 structure. Kaplan‐Meier analysis revealed that high TFAP2A expression might predict poor clinical prognosis in patients with lung cancer (Figure S6F, Supporting Information). Notably, *TFAP2A* and *ATF4* levels significantly increased under 0.25 mM glutamine stimulation (Figure S6G, Supporting Information). Inhibition of *TFAP2A* reduced *ATF4* expression and suppressed cancer cell proliferation in the presence of 0.25 mM glutamine (Figure S6H,I, Supporting Information). These findings collectively suggest that glutamine restriction upregulates TFAP2A expression levels, and TFAP2A directly binds to the *ATF4* promoter sequence to positively regulate ATF4 transcription.

### 2.6 COP Suppresses the GCN2‐ATF4‐ASCT2 Axis and the Downstream mTOR Signaling Pathway

**Figure 6:**
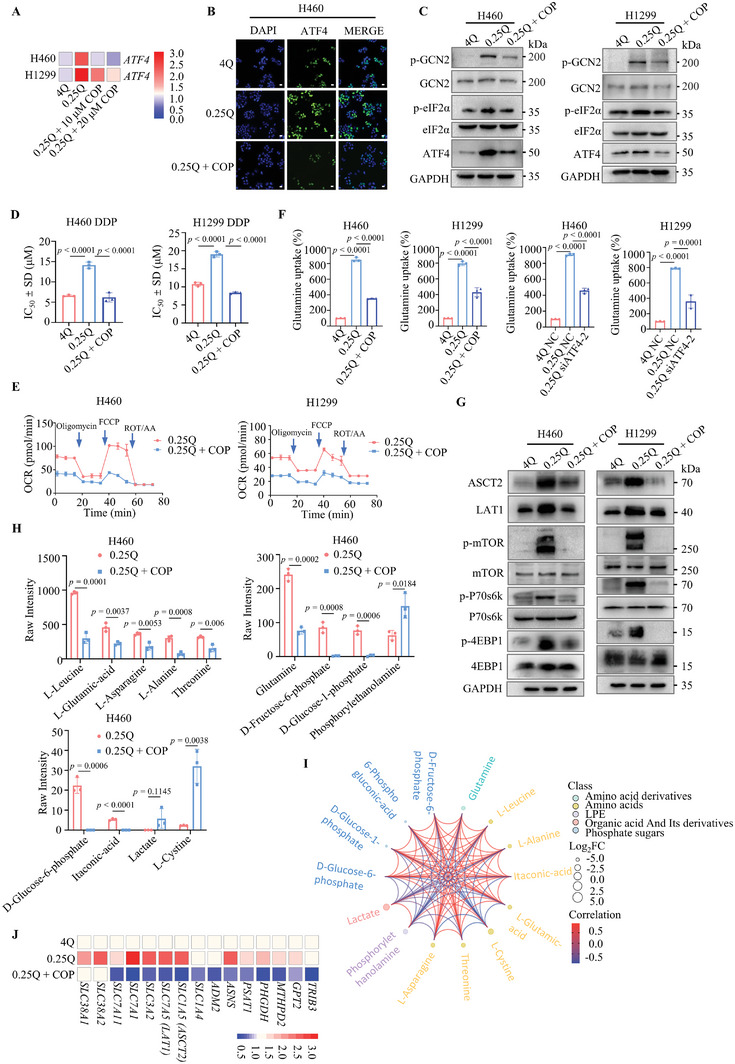
COP suppresses the GCN2‐ATF4‐ASCT2 axis and the downstream mTOR signaling pathway. A) qRT‐PCR analysis of *ATF4* mRNA level in NCI‐H460 or NCI‐H1299 cells after 24 h treatment with 0.25 mM glutamine or 0.25 mM glutamine plus COP (10 µM or 20 µM) (*n* = 3 independent experiments, average of three technical replicates). Heatmap colors represent 2^−ΔΔCt^ values. B) Effects of COP (10 µM) on NCI‐H460 stimulated with 0.25 mM glutamine analyzed by immunofluorescence (*n* = 3 independent experiments). Scale bar: 20 µm. C) Immunoblotting of GCN2 signaling analyses: phospho‐GCN2 (p‐GCN2, Thr899), total GCN2, phospho‐eIF2α (p‐eIF2α, Ser51), total eIF2α, and ATF4 in NCI‐H460 and NCI‐H1299 cells cultured in 4 mM or 0.25 mM glutamine conditions (12 h). D) NCI‐H460 and NCI‐H1299 cells treated with 4 mM or 0.25 mM glutamine for 3 days were switched to complete RPMI‐1640 medium for 2 h to induce persister cells. These persister cells were subsequently treated with COP (20 µM), and the IC_50_ of cisplatin was assessed in live cell plates following drug treatment. (*n* = 3 independent experiments). E) Seahorse XF assay measuring OCR of NCI‐H460 or NCI‐H1299 cells in 0.25 mM glutamine or 0.25 mM glutamine combined with COP (10 µM) (*n* = 3 independent experiments). F) Glutamine uptake rate of NCI‐H460 or NCI‐H1299 cells cultured in 4 mM or 0.25 mM glutamine in the absence or presence of COP (20 µM) for 36 h or ATF4 knockdown (*n* = 3 independent experiments). G) Immunoblotting of ASCT2, LAT1, phospho‐mTOR (p‐mTOR, S2448), total mTOR, phospho‐P70S6K (p‐P70S6K, Thr389), total P70S6K, phospho‐4EBP1 (p‐4EBP1, Thr37/46), total 4EBP1 protein levels in NCI‐H460 and NCI‐H1299 cells cultured in 4 mM or 0.25 mM glutamine conditions with or without COP treatment (20 µM) (48 h). H) Mass spectrometry analysis of differential metabolites in 0.25 mM glutamine and 0.25 mM glutamine plus 20 µM COP on H460 cells (*n* = 3 independent experiments). I) Correlation analysis of differential metabolites in 0.25 mM glutamine and 0.25 mM glutamine combined with COP (20 µM). The size of the point in the figure represents the Log_2_FC value, and the larger the point is, the larger the corresponding Log_2_FC value is, the color of the point represents the source classification of the differential metabolites in this group, and the connection represents the correlation coefficient value of the metabolite in the corresponding position. J) qRT‐PCR analysis of ATF4‐downstream transcripts in NCI‐H460 cells after COP (20 µM) treatment in 0.25 mM glutamine for 24 h (*n* = 3 independent experiments, average of three technical replicates). 4Q: 4 mM glutamine, 0.25Q: 0.25 mM glutamine, COP: coptisine chloride. All immunoblots are representative of three biological replicates that showed similar results. Data are shown as mean ± SD. *p < 0.05, **p < 0.01, ***p < 0.001, ****p < 0.0001. Data were analyzed by two‐tailed Student's t‐tests (H) and One‐way ANOVA (D, F) in GraphPad Prism 9.5.0.

The intriguing affinity of COP for G4 molecular structures piqued our interest in the further investigation of its biological activities. RNA‐Seq analysis revealed that the presence of COP decreased ATF4 expression under conditions of glutamine restriction (Figure S7A, Supporting Information). We demonstrated that COP significantly reduced the expression of ATF4 induced by glutamine restriction (**Figure** **6**A–C; Figure S7B,C, Supporting Information), leading to substantial inhibition of cancer cell proliferation (Figure S7D–F, Supporting Information). In PS induced with either 4 mM or 0.25 mM glutamine, the addition of COP resulted in a reduction of cisplatin resistance linked to glutamine deficiency (Figure 6D). Moreover, the OCR assay revealed that COP notably decreased mitochondrial respiration in NCI‐H460 and NCI‐H1299 cells (Figure 6E). Notably, the enhanced glutamine uptake and utilization in the context of glutamine deficiency was substantially hindered by COP treatment or ATF4 knockdown (Figure 6F).

Activation of GCN2 is observed under conditions involving amino acid stress, subsequently leading to the phosphorylation of eIF2α, a process utilized to suppress general mRNA translation while promoting ATF4 expression. Our investigation elucidated that the phosphorylation of GCN2 (Thr899) and eIF2α (Ser51) was triggered by glutamine‐nutritional restriction, subsequently driving the protein expression of ATF4. Importantly, this stress‐induced activation was attenuated by COP treatment (Figure 6C; Figure S7C, Supporting Information). Glutamine is transported into cells through the glutamine transporter (SLC1A5), which controls intracellular glutamine levels. Subsequently, SLC7A5 utilizes intracellular glutamine as an efflux substrate to modulate the uptake of extracellular leucine into cells.^[^ ^48^ ^]^ Previous studies have established that leucine regulates the mTORC1 signaling pathway in a variety of cellular processes to promote proliferation.^[^ ^49^ ^]^ Activation of GCN2 under glutamine deficiency triggers increased expression of the SLC1A5 transporter, facilitating the exchange of extracellular leucine and ultimately leading to mTORC1 signaling activation. mTOR is a conserved serine/threonine kinase that responds to changes in nutrient levels and growth signals.^[^ ^50^ ^]^ It acts as a catalytic subunit for two primary protein complexes: mTORC1, which is a key regulator of cell metabolism and growth, and mTORC2, which is crucial for controlling cell proliferation and survival. mTORC1 is a central signaling hub that integrates signals from nutrients, metabolic intermediates, and growth factors to regulate cellular metabolism in response to the environment.^[^ ^51^ ^]^ Our experiments demonstrated that the phosphorylation of mTORC1 and its downstream effectors P70S6K and 4EBP1 was activated by glutamine restriction, and this activation was inhibited in the presence of COP (Figure 6G; Figure S7G, Supporting Information). Collectively, these findings suggest that COP reduces ATF4 levels, thereby diminishing stress‐induced cancer cell survival.

To further validate the potential impact of COP, we conducted a metabolomic analysis on NCI‐H460 cells treated with different conditions: 4 mM glutamine, 4 mM glutamine plus COP, 0.25 mM glutamine, or 0.25 mM glutamine plus COP. Using the Energy Metabolism Database v2.0, we identified 68 metabolites and enriched metabolic pathways by analyzing the differential metabolites upon COP stimulation (Figure S8A, Supporting Information). Principal component analysis of metabolism revealed the COP‐induced downregulation of Glutamine, L‐Leucine, L‐Glutamic acid, and L‐Asparagine (Figure 6H). These findings suggest that COP not only further restrains amino acid breakdown and utilization in cancer cells but also heightens sensitivity to glutamine deprivation. Using Pearson's correlation analysis, we assessed the correlation between metabolites with significant differences (Figure 6I). Additionally, to observe changes in metabolite trends across various samples, we standardized and centralized the relative contents of distinct metabolites and subjected them to K‐means clustering analysis. Remarkably, the inclusion of COP significantly affected the TCA cycle and the pentose phosphate pathway (Figure S8B, Supporting Information).

Quantitative real‐time polymerase chain reaction (qRT‐PCR) was employed to gauge changes in downstream target genes of *ATF4* in NCI‐H460 cells (Figure 6J). These results indicated that the expression of genes downstream of *ATF4* was upregulated under glutamine deficiency and subsequently downregulated upon COP treatment. In addition, COP exerted a notable inhibitory effect on the amino acid transporter *ASCT2*. Findings from colony formation and EdU assays showed that *ASCT2* knockdown markedly intensified cancer cell proliferation (Figure S9A–E, Supporting Information). The efficacy of ASCT2 inhibition in curbing glutamine uptake has been demonstrated across various cancer types, including melanoma,^[^ ^52^ ^]^ non‐small cell lung cancer,^[^ ^53^ ^]^ prostate cancer,^[^ ^54^ ^]^ and acute myeloid leukemia.^[^ ^55^ ^]^ Kaplan‐Meier plots, coupled with the log‐rank (Mantel‐Cox) test highlighted the prognostic benefits associated with low ASCT2 expression in NSCLC (Figure S9F, Supporting Information).

### 2.7 COP Decreases Glutamine Deficiency‐Induced ATF4 Levels and Improves the Efficacy of Glutamine‐Restrictive Therapy

**Figure 7:**
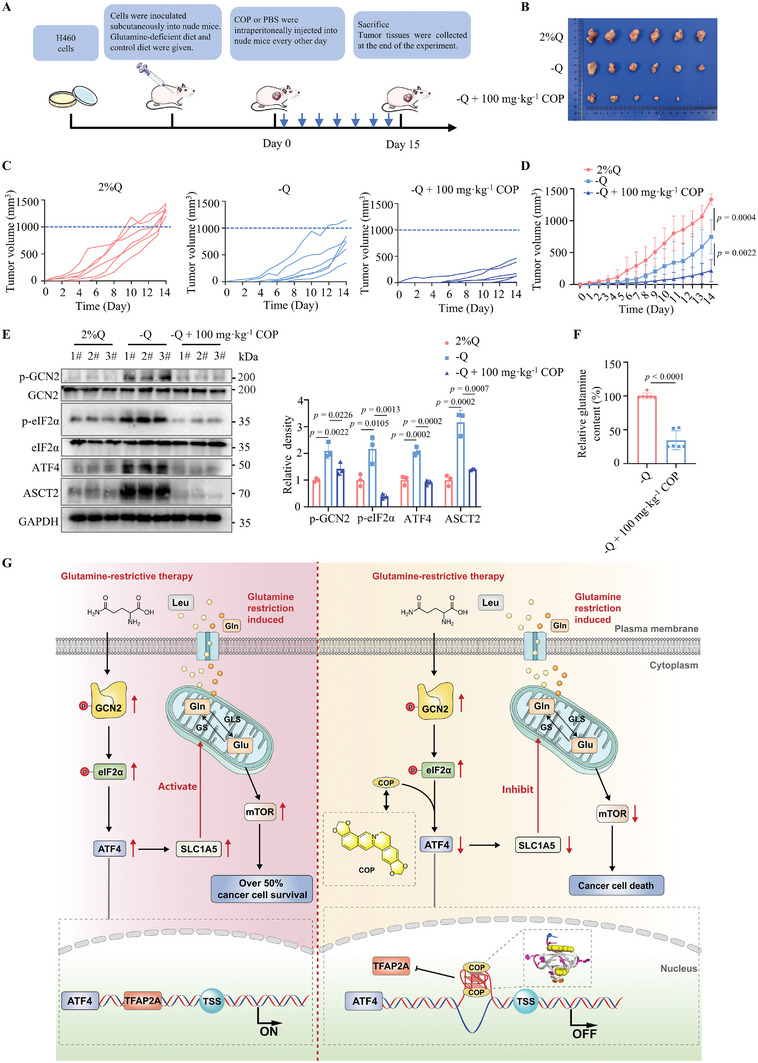
COP decreases glutamine deficiency‐induced ATF4 levels and improves the efficacy of glutamine‐restrictive therapy. A) Protocol of COP (100 mg·kg^−1^) administration in glutamine restriction therapy and establishment of H460 xenograft tumor model. B) Image of tumor removal after intraperitoneal injection of COP (100 mg·kg^−1^) (*n* = 6 per group). C) Growth curves of tumor volume (*n* = 6 per group). D) Tumor volume of nude mice with COP treatment and glutamine restriction diet (*n* = 6 per group). E) Immunoblotting of indicated proteins in xenograft tumors after COP and glutamine restriction diet therapy in vivo (*n* = 3 independent experiments). F) Determination of glutamine content in xenograft tumors with glutamine restriction (*n* = 6 independent experiments). G) Proposed model of COP targeting *ATF4‐*G4 enhancing sensitivity to glutamine restriction therapy. All immunoblots are representative of three biological replicates that showed similar results. Glutamine concentrations of 2% and 0% glutamine diet are represented by 2%Q and ‐Q, respectively. COP: coptisine chloride. Data are shown as mean ± SD. *p < 0.05, **p < 0.01, ***p < 0.001, ****p < 0.0001. Data were analyzed by two‐tailed Student's *t*‐tests (D,F) and One‐way ANOVA (E) in GraphPad Prism 9.5.0.

To assess the effects of COP in vivo, we conducted xenograft experiments under both normal (with adequate glutamine) and glutamine‐deficient conditions (**Figure** **7**A). Compared to normal conditions, xenograft growth was delayed owing to glutamine restriction (Figure 7B,C). Remarkably, COP significantly enhanced the inhibitory effect of glutamine‐restrictive therapy on tumor growth (Figure 7D; Figure S10A,B, Supporting Information). Western blot analysis demonstrated that COP effectively disrupted the GCN2‐ATF4‐ASCT2 axis (Figure 7E). Furthermore, IHC analysis revealed that COP diminished the expression of ATF4 and ASCT2 during glutamine restriction (Figure S10C, Supporting Information), thereby influencing the uptake and utilization of glutamine by ASCT2 within tumor tissues during glutamine deficiency (Figure 7F). Collectively, these findings highlight the ability of COP to augment tumor responsiveness to dietary glutamine restriction, offering a novel therapeutic avenue for cancer treatment.

As illustrated in Figure 7G, glutamine restriction triggered ATF4 translational reprogramming, which accounted for over 50% of persister cancer cell survival and weakened the efficacy of glutamine‐deficient therapy. Our study demonstrates that the natural compound COP targets the *ATF4‐*G4 structure, disrupts the interaction between TFAP2A and the *ATF4* promoter, and curtails glutamine uptake and utilization by inhibiting the *ATF4* downstream gene *SLC1A5*. This cascade of events subsequently suppresses the mTOR signaling pathway, ultimately enhancing the effectiveness of glutamine‐restrictive therapy.

## 3 Discussion

PS represents a nongenetically reversible state and exhibits a slow‐cycling phenotype in cancer.^[^ ^1^ ^]^ Cancer relapse begins when malignant cells experience the extreme metabolic stress caused by chemotherapy.^[^ ^11^ ^]^ Metabolic networks provide cells with stress protection systems. Exploiting limited amino acid metabolism to selectively target highly proliferative cancer cells has gained substantial attention in recent years.^[^ ^56^ ^]^ Tumor growth and survival are intricately linked to the nutrients obtained from the host. Modifications in the host diet can potentially alter nutritional availability within the tumor microenvironment, offering a promising approach to impede tumor growth. Amino acid restriction orchestrates various genetic expression steps including chromatin configuration, transcription initiation sites, transcription rates, mRNA splicing, RNA export, RNA turnover, and translation, all of which ultimately govern the DNA‐to‐RNA‐to‐protein cascade.^[^ ^6b^ ^]^ These promising results suggest that amino acid restriction strategies are potential rational therapeutic interventions for nutrient‐dependent persister cancer cells. However, dietary protein restriction triggers the activation of metabolic reprogramming pathways aimed at restoring cellular amino acid equilibrium, which poses a significant challenge for the effective clinical translation of amino acid restriction methodologies.^[^ ^57^ ^]^ Our research revealed that under glutamine nutritional restriction, the ISR component, GCN2, was activated. This activation led to the phosphorylation of translation initiation factor eIF2α,^[^ ^58^ ^]^ subsequently triggering the activation of ATF4. Therefore, targeting ATF4 is a promising strategy for enhancing the persistent cellular sensitivity to glutamine restriction therapy.

The *ATF4* promoter contains a unique nucleic acid secondary structure known as G4, which functions as a negative transcriptional modulator susceptible to intervention by small molecules. However, the lack of a well‐established complex structure involving *ATF4‐*G4 and its ligand has impeded progress in targeting key factors in glutamine restriction therapy for NSCLC. In this context, we present the NMR solution structures of *ATF4‐*G4 bound to COP. The resulting complex structure revealed a 2:1 binding stoichiometry, with each compound engaging the flanking adenine residue to create a “quasi‐triad plane” that intercalated between the two external G‐tetrads. This binding mechanism involved both π‐stacking and electrostatic interactions. Furthermore, COP significantly reduced *ATF4* mRNA levels in cancer cells. Mechanistically, COP targets ATF4 to curtail glutamine uptake and utilization in NSCLC, concurrently inhibiting the mTOR signaling pathway. The revelation of COP interaction with the *ATF4‐*G4 structure advances research on glutamine restriction therapy for NSCLC. Our study provides valuable insights into ligand interactions with *ATF4‐*G4 and paves the way for targeting the *ATF4*‐G4 structure to impede tumor growth, which is a critical aspect in the development of drugs that interact with *ATF4‐*G4.

The concept of “starving cancer to death” is based on the notion that numerous metabolic changes within cancer cells offer potential vulnerabilities that can be targeted.^[^ ^59^ ^]^ Our metabolic analysis of NCI‐H460 cancer cells revealed significant changes in the amino acid profile of NSCLC cells in response to glutamine nutritional restriction, which reduced the reliance of NSCLC cells on glutamine addiction. The multifaceted role of glutamine in tumor progression, coupled with its efficacy in inhibiting tumor growth across diverse cancer types in vitro and in vivo,^[^ ^60^ ^]^ has been extensively demonstrated. Presently, dietary amino acid manipulation involving either amino acid deprivation or supplementation is a promising avenue for overcoming chemotherapy resistance and halting cancer progression.^[^ ^61^ ^]^ In various melanoma xenograft models, dietary glutamine supplementation led to heightened α‐ketoglutarate‐dependent hypomethylation, desensitizing BRAF inhibitor‐resistant cells.^[^ ^62^ ^]^ Cisplatin‐resistant NSCLC cells exhibit increased extracellular glutamine uptake and enhanced glutaminase activity, rendering them susceptible to glutamine deprivation.^[^ ^63^ ^]^ Modulation of glutamine metabolism targets transcription activating factors (STAT) 1/3, reducing IDO gene expression within tumor cells and enhancing the effectiveness of anti‐cancer T cells,^[^ ^64^ ^]^ thereby rejuvenating checkpoint blockade therapy in drug‐resistant tumors. The growing recognition of nutrient‐based strategies that exploit tumor metabolic vulnerabilities underscores their potential efficacy in curbing cancer nutrition. However, universal dietary composition recommendations are unlikely to emerge for cancer prevention and treatment. Instead, combining chemotherapeutic agents with targeted amino acid therapies has the potential to combat drug‐resistant cancers driven by amino acid metabolism. Similar to novel drug combinations, a personalized approach involving medication and dietary adjustments is crucial for each cancer type, location, and grade. Our research demonstrates that the natural compound COP enhances cancer sensitivity to glutamine restriction by specifically targeting *ATF4‐*G4, a pivotal component of the ISR signaling pathway that is activated in response to glutamine deficiency. We explored the distinct metabolic attributes associated with elevated ATF4 levels under glutamine restriction and COP treatment. Our findings also revealed that TFAP2A bound to the *ATF4* promoter region. This interaction was observed at the G4 formation site within the *ATF4* promoter, ultimately promoting the positive modulation of ATF4 expression.

In conclusion, our study provides insights into the therapeutic potential of selectively targeting *ATF4‐*G4 using small molecules to enhance the glutamine restriction strategy. The findings offer a new avenue for the elimination of PS in glutamine restriction therapy.

## 4 Experimental Section

### Reagents and Cell Culture

The cell lines utilized in this study were obtained from the National Collection of Authenticated Cell Cultures (Shanghai), and included NCI‐H460, NCI‐H1299, NCI‐H1975, NCI‐HCC827, NCI‐H661, A549, and PC‐9. These cell lines were cultured in RPMI 1640 medium (Gibco, 11875‐093), supplemented with 10% fetal bovine serum (ExCell Bio, FCS500) and 100 U/ml penicillin‐streptomycin (New Cell and Molecular Biotech, China; C100C5). The cells were maintained at 37 °C in a humidified atmosphere composed of 95% air and 5% CO_2_. Regular testing was conducted to ensure the absence of Mycoplasma contamination. Following attachment, the cells were treated with conditioned medium containing varying concentrations of glutamine (4 mM, 0.5 mM, 0.25 mM, and 0 mM glutamine).

DNA oligonucleotides were obtained from Sangon Biotech (Shanghai) Co., Ltd. and were available in two different purity grades: HPLC and PAGE. The DNA was solubilized in a buffer containing 37.5 mM KCl and 12.5 mM K_2_HPO_4_/KH_2_PO_4_, pH 7, with a D_2_O/H_2_O ratio of 10/90. Final concentrations were determined using a UV spectrometer. The sequences of the oligonucleotides are listed in Table S3 (Supporting Information). The compound coptisine chloride (COP) (CAS NO. 6020‐18‐4) was purchased from Shanghai Standard Technology Co., Ltd. The GCN2 inhibitor GCN2iB (CAS NO. 2183470‐12‐2) was obtained from Med Chem Express (America).

### Generation of Persister Cells

NCI‐H460 and NCI‐H1299 cells treated with 4 mM or 0.25 mM glutamine for 1–5 days were switched to complete RPMI‐1640 medium for 2 h to create persister cells.^[^ ^65^ ^]^ Live cells were sorted using the Dead Cell Removal Kit (Miltenyi Biotec, Germany). The Dead Cell Removal MicroBeads target a moiety in the plasma membrane of both apoptotic and dead cells. To deplete dead cells, cells were magnetically labeled with Dead Cell Removal MicroBeads and passed through a separation column. The magnetically labeled dead cells were captured within the column, while the unlabeled living cells passed through, resulting in a cell fraction free of dead cells. NCI‐H460 and NCI‐H1299 cells were counted after treatment with 4 mM or 0.25 mM glutamine. The cell suspension was centrifuged at 300×g for 10 minutes, followed by complete aspiration of the supernatant. The cell pellet was then resuspended in 100 µL of Dead Cell Removal MicroBeads per 10⁷ total cells, mixed well, and incubated for 15 min at room temperature (20 to 25 °C). 1× Binding Buffer was added to the cell suspension to achieve a minimum volume of 500 µL for separation. Magnetic separation was then carried out by placing the column in the magnetic field of a suitable MACS Separator, rinsing the column with the appropriate amount of 1× Binding Buffer, applying the cell suspension onto the column, and collecting the flow‐through containing unlabeled cells (live cells). The column was washed with the appropriate amount of 1× Binding Buffer and mixed thoroughly. The screened live cells were then centrifuged, resuspended in a medium containing 4 mM and 0.25 mM glutamine for counting, and finally seeded into plates.

### Cell Viability Assay

Cell viabilities were assessed using the Cell Counting Kit‐8 (Med Chem Express, America). In brief, cells were seeded onto 96‐well plates at a density of 2 × 10^3^ per well and exposed to varying concentrations of COP or conditioned medium. The plates were then maintained at 37°C for 24, 48, and 72 h. Subsequently, 10 µL of CCK‐8 reagent (diluted in 100 µL medium per well) was added to the cells, and they were incubated for 2 h at 37 °C in a 5% CO_2_ incubator. The absorbance at a wavelength of 450 nm was measured using a microplate reader. This assay was repeated at least three times, with triplicate measurements each time. For the trypan blue exclusion assay, 2 × 10^5^ cells were cultured in complete medium in 60 mm^3^ dishes and incubated at 37 °C. The cells were treated with conditioned medium (4 mM glutamine, 0.25 mM glutamine) and the appropriate culture medium for 1 to 5 days, starting from the second day. After preparing a cell suspension, the suspension was mixed with 0.4% trypan blue at a 9:1 ratio (final concentration 0.04%). The mixture was then stained for 3 min, and the viable cells were counted. In the ethynyldeoxyuridine (EdU) analysis, 1.2 × 10^4^ cells were seeded in 96‐well plates and exposed to EdU for a duration of 4 h. The subsequent steps were performed according to the manufacturer's protocol. The results were captured using the Molecular Devices ImageXpress High Content Confocal Imaging System and quantified using Image J. For the cell apoptosis experiment, cells were treated with conditioned medium (4 mM glutamine, 0.25 mM glutamine) for 24 h. After fixation, the cells were stained with Hoechst 33 342 (1000×) and visualized using a fluorescent microscope.

### Colony Formation Assay

A range of 2000 to 4000 cells per well were seeded into 6‐well plates with complete growth medium and allowed to settle overnight. On the following day, the cells were treated with conditioned medium for a duration of 7 to 12 days, during which visible colonies formed. For NCI‐H460 and NCI‐H1299 cells, transfection was carried out using shScramble, shATF4, pHBLV‐CMV‐MCS‐3Flag‐ZsGreen‐T2A‐PURO vector, and pHBLV‐CMV‐ATF4‐3Flag‐ZsGreen‐T2A‐PURO plasmids (HANBIO, China) in combination with Lipofectamine 3000 (Thermo Fisher Scientific). Post‐transfection, cells were plated into 6‐well plates with 2000 cells per well, following the previously described protocol. The resultant colonies were washed with phosphate‐buffered saline (PBS) and fixed using 4% Paraformaldehyde Fix Solution (Servicebio, G1101‐500ML) for 10 min at room temperature. Subsequently, they were stained with Crystal Violet Staining Solution (Beyotime, C0121‐100ML) for 10 min. After a PBS wash, colony counting was performed for statistical analysis.

### RNA Extraction and Quantitative RT‐PCR

Total RNA was extracted using the RNA extraction kit (Shanghaiyishan, China) following the manufacturer's instructions. Subsequently, cDNA was synthesized using the HiScript Q RT SuperMix kit (Vazyme, China). Quantitative RT‐PCR analyses were conducted on the LightCycler 480 real‐time fluorescent quantitative PCR system (Roche, Germany). The threshold cycles (Ct values) of the target genes were normalized to GAPDH, which was used as the endogenous control. All qPCR amplifications were carried out in triplicate and repeated in three independent experimental runs. The primer sequences utilized for qPCR can be found in Table S1 (Supporting Information).

### Measurement of ROS Production

Cells were exposed to conditioned medium, followed by trypsinization and collection into 1.5 mL tubes. After collection, the cells were resuspended in a diluted solution of DCFH‐DA and incubated at 37°C for 20 min. Subsequently, the cells underwent three washes using serum‐free cell culture medium to remove any residual DCFH‐DA that had not entered the cells. The detection procedure was conducted using a BD Caliber flow cytometer (BD Biosciences), and the results were analyzed using FlowJo software.

### Western Blot Analysis

Total proteins were extracted from cells or tumor tissue homogenates using NP‐40 lysis buffer. The protein samples were then separated by SDS‐PAGE gel and transferred onto polyvinylidene fluoride (PVDF) membranes (Bio‐Rad, CA, USA). Subsequent to transfer, these membranes were incubated with specific antibodies, including the following primary antibodies: ASCT2 (Proteintech, 20350‐1‐AP, 1:2000), HRI (Proteintech, 20499‐1‐AP, 1:1000), TFAP2A (Proteintech, 13019‐3‐AP, 1:2000), p‐P70(S6K) (Thr389) (Proteintech, 28735‐1‐AP, 1:2000), P70(S6K) (Proteintech, 14485‐1‐AP, 1:2000), SLC7A5 (Proteintech, 28670‐1‐AP, 1:2000), ATF4 (Abcam, ab184909, 1:500), mTOR (Abcam, ab13a4903, 1:10000), p‐mTOR (S2448) (Abcam, ab109268, 1:1000), GCN2 (Abcam, ab134053, 1:1000), eIF4EBP1 (Abcam, ab32024, 1:2000), p‐GCN2 (Thr899) (Affinity, AF8154, 1:1000), p‐4EBP1 (Thr37/46) (Cell Signaling Technology, 2855, 1:1000), eIF2α (Cell Signaling Technology, 5324, 1:1000), p‐eIF2α (Ser51) (Cell Signaling Technology, 3398, 1:1000), GAPDH (Cell Signaling Technology, 97 166, 1:1000). Following incubation with primary antibodies, immunocomplexes were visualized through a chemiluminescence reaction using ECL reagents (Vazyme, China) and quantified using Image J software. The antibodies employed for western blotting are listed in Table S4 (Supporting Information).

### Glutamine Uptake Assay

Cells were initially seeded in 100 mm^3^ tissue culture plates with complete growth medium and incubated overnight. The following day, cells were harvested after 36 h of treatment with conditioned medium. For tissue samples, cells were lysed using established protocols, and each group was then normalized based on protein quantification. To quantify glutamine uptake, a glutamine assay kit (ab197011, Abcam) was used, following the manufacturer's instructions. This experiment was repeated three times and conducted in three independent iterations.

### Seahorse Analysis

The cellular oxygen consumption rate (OCR) was measured using the Seahorse XFe96 Analyzer (Agilent Technologies). Cells treated with COP and knock‐down ATF4 were resuspended in Seahorse XF RPMI 1640 medium (supplemented with 10 mM glucose, 1 mM pyruvate, and 2 mM glutamine), and seeded in Cell‐Tak (Corning) precoated Seahorse 96‐well plates. The OCR was assessed under basal conditions and in response to 1.5 µM oligomycin, 1.5 µM fluorocarbonyl cyanide phenylhydrazone (FCCP), 0.5 µM rotenone, and antimycin A.

### Immunofluorescence

After the cellular pre‐treatment, cells were fixed in a 4% paraformaldehyde solution for 20 min. Subsequently, cells were permeabilized with 0.1% Triton X‐100 for 10 min, followed by blocking with a 0.5% bovine serum albumin solution for 2 h at room temperature. In the next step, samples were subjected to an overnight incubation with primary antibodies, followed by an additional incubation with fluorescently conjugated secondary antibodies and DAPI. Captured images were acquired using the ImageXpress Micro system, and quantification was performed using Image J software. Immunofluorescence antibodies are listed in Table S4.

### NMR Spectroscopy Experiments

NMR spectra were acquired using a Bruker AV‐600 spectrometer equipped with a QCI Cryoprobe. The w5 water peak suppression was configured for the entire spectrum collection process. NMR spectra processing was performed using Topspin 3.5 (Bruker) and Sparky (UCSF) software. 2D NOESY experiments were conducted with varying mixing times of 80, 300, 350, and 400 ms, at temperatures spanning 5, 10, 15, 25, and 35 °C in a solvent mixture of 90% H_2_O/10% D_2_O. The DQF‐COSY spectrum was acquired at a temperature of 25 °C. ^1^H‐^13^C HSQC spectra were obtained using the hsqcetgpsi pulse sequence with a ^1^J (C, H) coupling constant of 145 Hz.

### Structure Calculation

NOE‐based distance restraints were categorized into different strengths: strong (2.9 ± 1.1 Å), medium (4.0 ± 1.5 Å), weak (5.5 ± 1.5 Å), and very weak (6.0 ± 1.5 Å), based on the NOESY spectra collected with mixing times of 80 and 350 ms at temperatures of 25 and 5 °C, respectively. Exchangeable protons were assigned corresponding to medium (4.0 ± 1.2 Å), weak (5.0 ± 1.2 Å), and very weak (6.0 ± 1.2 Å) distances. Intermolecular cross‐peaks between COP and DNA were defined by very weak (6.0 ± 1.5 Å), weak (5.0 ± 1.5 Å), and medium (4.0 ± 1.5 Å) distances. Overlapping and ambiguous resonances were categorized as having a distance of 5.0 ± 2.0 Å.

For the 12 tetrad guanines, 48 hydrogen bond restraints were applied. Dihedral restraints were employed for glycosidic torsion angles with values of 170°−310° and 200°−280°, corresponding to anti‐conformations in loop regions and within the G‐tetrad, respectively. The structure calculation incorporating NOE restraints was carried out using Xplor‐NIH74 in conjunction with the Amber 20 package. An ensemble of 100 starting structures was generated using Xplor‐NIH. COP parameter files were initially obtained from ChemDraw18.2 and were further optimized and computed using the Gaussian 09 program. Subsequently, the 100 initial structures underwent simulated annealing using the sander module of the Amber 20 package. The 20 structures with the lowest energy were selected for molecular dynamic simulations with the pmemd module of the Amber 20 package, in the presence of K^+^ cations and TIP3P water. Finally, the last 500 ps of trajectories were averaged, followed by energy minimization for 500 steps in a vacuum, after removal of water molecules and cations. The final ensemble consisted of the 10 lowest‐energy structures, which were chosen for deposition and analysis. Visualization and analysis were performed using VMD and PyMOL software.

### CD Experiments

CD data were acquired using a Jasco‐1500 spectropolarimeter (Jasco Inc., Japan). DNA samples were prepared in a buffer containing 5 mM K_2_HPO_4_/KH_2_PO_4_ at pH 7, with a final concentration of 20 µM. The DNA samples were annealed at 95 °C for 5 min and then gradually cooled to room temperature using a heating block. Subsequently, the desired concentration of COP was added, and the mixture was incubated for 2 h. CD experiments were performed in a 1 mm path length quartz cuvette at a temperature of 25 °C. A baseline measurement was obtained using the buffer spectrum. To account for any blank, the baseline was subtracted, and the Savitzky‐Golay smoothing algorithm was applied to enhance the spectral smoothness. For CD melting analysis, the sample was heated from 25 to 95 °C at a heating rate of 2 °C·min^−1^, while monitoring the CD ellipticity at 264 nm. The melting temperature was determined at the point where the median of the fitted baselines intersected with the melting curve.

### Fluorescence Measurements

Fluorescence measurements were performed using a Jasco‐FP8300 spectrofluorometer (Jasco Inc., Japan). The fluorescence spectra were recorded using a 1 cm path length quartz cell. Emission spectra were captured within the range of 520 to 600 nm, with an excitation wavelength set at 377 nm. COP concentrations were held at 0.2 µM in a 50 mM K^+^‐containing solution, with a total volume of 3 mL. DNA was incrementally introduced at the desired concentration. Following a 2‐min incubation period at each step, fluorescence spectra were collected.

### Native Electrophoretic Mobility Shift Assay (EMSA)

EMSA gels were prepared using a 10 × 7 cm native gel with a thickness of 1 mm, containing 12.5 mM KCl and 16% acrylamide (acrylamide/bisacrylamide ratio of 29:1), pH 8.0. DNA samples were dissolved in a 5 mM KCl, 12.5 mM phosphate buffer at pH 7.0. COP was added at the desired concentration and incubated for 2 h. Electrophoresis was carried out at a voltage of 45 V in 1 × TBE running buffer. DNA bands were visualized at 260 nm using UV light.

### DMS Footprinting

DMS footprinting was conducted following the described protocol with certain modifications.^[^ ^39b^ ^]^ DNA samples were annealed in the presence of 50 mM KCl or LiCl and subsequently treated with 0.4% DMS (final concentration) for 2 min at room temperature in a total volume of 200 µL. The methylation reaction was promptly stopped by adding 150 µL of freshly prepared stop buffer containing 2.5 M NH_4_OAc, 0.1 M β‐mercaptoethanol, and 1 mg·mL^−1^ sperm DNA. Following phenol/chloroform extraction and ethanol precipitation, the DNA was reconstituted in water with the addition of 100 µL of 10% (vol/vol) piperidine in water and then heated at 90 °C for 30 min to cleave the methylated DNA. Subsequently, chloroform extraction and ethanol precipitation were performed. The precipitated DNA was dissolved in 80% (vol/vol) deionized formamide in water, denatured at 95 °C for 5 min, and separated on a 20% denaturing polyacrylamide gel. DNA fragments were visualized using a FAM dye covalently labeled at the 5′‐end of the DNA on a GelGo Imager (BIO‐RAD, USA) and digitized using the ImageQuant 5.2 software.

### ChIP

NCI‐H1299 cells in the logarithmic growth phase were seeded into sterile cell culture dishes at a density of 1 × 10^6^ cells per dish. Once adhered, the medium was exchanged with either 4 mM glutamine, 0.25 mM glutamine, or 0.25 mM glutamine supplemented with 20 µM COP. After a 12‐h treatment period, the cells were cross‐linked using 1% formaldehyde at room temperature for 10–15 min. Following centrifugation, the cells were lysed using Lysis Buffer (1 × 10^6^ cells / 200 µL) on ice for 10 min. Subsequently, the cells were resuspended in ChIP buffer (1 × 10^6^ cells / 100 µL) and subjected to ultrasonic treatment at 25% power for 3–4 pulses to facilitate chromatin extraction. DNA shearing was verified via agarose gel electrophoresis. The ChIP reaction system was prepared, followed by reverse cross‐linking and DNA release. DNA purification and qRT‐PCR reactions were performed. The enrichment rate was calculated using the formula FE% = 2^−ΔΔCt^ × 100, where ΔΔCt = (Ct target gene – Ct IgG) in the treatment group – (Ct target gene – Ct IgG) in the untreated group. The Chroma Flash High‐Sensitivity ChIP Kit (EpigenTek) was employed following the manufacturer's instructions. The experiment was carried out in triplicate and replicated three times. Primers used for ChIP are listed in Table S2 (Supporting Information).

### RNA‐Seq

Three distinct sample groups (4Q, 0.25Q, 0.25Q + COP) underwent total RNA extraction, rRNA removal, and mRNA enrichment using conventional kits. The enriched mRNA was reverse transcribed into double‐stranded cDNA, followed by repair of the cDNA ends, addition of junctions, and PCR amplification to generate on‐board libraries. Subsequently, RNA library sequencing was performed on the Illumina HiseqTM 2500/4000 by Gene Denovo Biotechnology Co., Ltd (Guangzhou, China). Bioinformatic analysis was performed using Omicsmart, a real‐time interactive online platform for data analysis available at http://www.omicsmart.com.

### Energy Metabolism Analysis

Metabolic profiling was performed on four distinct sample groups using liquid chromatography tandem mass spectrometry (LC‐MS/MS). Sample pre‐treatment was followed by machine analysis, with the liquid phase conditions as follows: The chromatographic column employed was ACQUITY UPLC BEH Amide (1.7 µm, 100 mm × 2.1 mm i.d.), maintained at a constant temperature of 40 °C. Phase A consisted of ultrapure water containing 10 mM ammonium acetate and 0.3% ammonia water, while phase B comprised a mixture of 90% acetonitrile and water. The flow rate was set at 0.40 mL·min^−1^, with an injection volume of 2 µL. The linear gradient elution profile was as follows: 0–1.2 min A/B at a ratio of 5:95 (V/V), 8 min A/B at 30:70 (V/V), 9–11 min A/B at 50:50 (V/V), and 11.1‐15 min A/B at 5:95 (V/V). The mass spectrometry conditions were set as follows: The spray ion source (ESI) temperature was adjusted to 550 °C. The mass spectrometry voltage in positive ion mode was set to 5500 V, while in negative ion mode, it was set to ‐4500 V. The Curtain Gas (CUR) pressure was maintained at 35 psi. Within the Q‐Trap 6500^+^, each ion pair was scanned and detected based on optimized settings for declustering potential (DP) and collision energy (CE). Quantitative analysis was carried out using multiple reaction monitoring (MRM) mode on the triple quadrupole mass spectrometry system. The mass spectrometry data were processed using Analyst 1.6.3 software and MultiQuant 3.0.3 software.

### Xenograft Tumor Assay

The animal experimental protocol underwent examination and approval by the Institutional Animal Care and Use Committee (IACUC) of the China Pharmaceutical University Experimental Animal Center (2023‐02‐015). Female BALB/c nude mice aged four to five weeks were sourced from Shanghai Sippr‐BK Laboratory Animal Co, Ltd. The mice were provided with both normal and glutamine‐deficient diets. A total of 3 × 10^6^ NCI‐H460 cells, suspended in 200 µL of PBS, were subcutaneously injected into the right flank of the mice. Once the xenograft volumes reached approximately 100 mm^3^, the mice were randomly assigned to their respective groups: Control (1% DMSO in cyclodextrin solution, normal diet), COP group (50 mg·kg^−1^ and 100 mg·kg^−1^ in cyclodextrin solution, normal diet), glutamine‐deficient feed group (1% DMSO in cyclodextrin solution, glutamine‐deficient diet), and the combination of glutamine‐deficient feed and COP group (50 mg·kg^−1^ in cyclodextrin solution, glutamine‐deficient diet) (*n* = 6 mice per group). Intraperitoneal (i.p.) injections of the compound were administered every other day for a span of two weeks. The tumor volume (mm^3^) of the mice was measured daily using calipers and calculated using the formula (length × width^2^)/2. Mice were euthanized via intraperitoneal injection of pentobarbital sodium. The xenografts were dissected, photographed, weighed, and fixed in 4% paraformaldehyde. For the intratumoral injection of shATF4 lentivirus, the mice were also randomly assigned to specific groups: Control (intratumoral injection of scramble control shRNA lentivirus, normal diet), shATF4 group (intratumoral injection of shATF4 lentivirus, normal diet), glutamine‐deficient feed group (intratumoral injection of scramble control shRNA lentivirus, glutamine‐deficient diet), and the combination of glutamine‐deficient feed and shATF4 group (intratumoral injection of shATF4 lentivirus, glutamine‐deficient diet). The lentivirus titer used was 1.0 × 10^9^ TU·mL^−1^ (shATF4 lentivirus: 5′‐GTTGGATGACACTTGTGAT‐3′, scramble control shRNA lentivirus: 5′‐TTCTCCGAACGTGTCACGTAA‐3′). Each tumor was injected at three points with 10 µL per point. Injections were administered every four days, totaling three injections. Following the injections, the normal feed group collected tumors on the 12th day, while the glutamine‐deficient group collected tumors on the 18th day. GraphPad Prism software was utilized to assess tumor volume, weight, and the weight of the mice. All analyses were conducted by individuals who were blinded to the experimental groups.

### Immunohistochemistry (IHC)

Immunohistochemistry (IHC) was performed following a standardized protocol. NSCLC tumor tissues were retrieved from mice, embedded in paraffin, and then sectioned into 3 µm slices on glass slides. The tissue sections underwent deparaffinization and rehydration. Primary antibodies were incubated at 4 °C overnight following antigen retrieval. The primary antibodies employed in this study were as follows: ATF4 (Abcam, ab184909, 1:100), ASCT2 (Proteintech, 20350‐1‐AP, 1:400), and Ki67 (Cell Signaling Technology, 9449, 1:800). Subsequently, the sections were exposed to fluorescence‐labeled secondary antibodies. Images were captured using a customized Nikon Eclipse C1 fluorescence microscope. Immunohistochemistry antibody details are listed in Table S4 (Supporting Information).

### Tissue Microarray Analysis

The NSCLC tissue microarray (AF‐LucSur2201) was collected and processed by AiFang Biological, and approved by the Institutional Review Board of Shanghai Pulmonary Hospital (K23‐334). In this study, tissue immunofluorescence was employed to evaluate the expression and colocalization of GS, GLS, and ATF4. Initially, the tissue microarray underwent rehydration, antigen retrieval, and blocking with peroxidase (3% hydrogen peroxide solution) and serum. Subsequently, the tissue microarray was incubated with anti‐ATF4 (Abcam, ab184909, 1:200), GS (Affinity, DF7341, 1:200), and GLS (Proteintech, 23549‐1‐AP, 1:200) antibodies overnight at 4 °C. Following this, horseradish peroxidase (HRP)‐labeled secondary antibodies were applied, and DAPI was used to restain the nuclei. Images were captured using a Nikon Eclipse C1 fluorescence microscope. Fluorescence quantification was performed by Wuhan Servicebio Biotechnology Co., Ltd using Aipathwell software.

### Statistical Analysis

Statistical analysis and graphical representation were performed using GraphPad Prism 9.5.0. The results are presented as means ± standard deviation (SD). All experiments were conducted independently at least three times. Data preprocessing for western blot and immunofluorescence quantification involved normalized quantification. A two‐tailed Student's t‐test was utilized to compare two groups, whereas one‐way analysis of variance (ANOVA) was employed for comparisons among multiple groups. Correlations were calculated using Pearson's correlation coefficient test. Survival curves were evaluated with Kaplan‐Meier plots and analyzed using logarithmic rank (Mantel‐Cox) tests. Statistical significance was defined as p values below 0.05, with “ns” representing non‐significant results.

## Conflict of Interest

The authors declare no conflict of interest.

## Author Contributions

C.X., Y.L., and Y.L. contributed equally to this work. C.X. performed most of the pharmacological experiments. Y.L. and Y.L. performed the G‐quadruplex related studies, particularly the NMR solution structure studies. C.X., Y.L., and Y.L. jointly analyzed the data, and wrote the original draft of the manuscript. K.W., Y.X., and L.K. initiated the project and made significant revisions to the manuscript. R.D. and X.H. participated in experimental method design and animal experiments. Q.L. and X.Z. performed the data statistics. All authors contributed to data analysis, drafting, and revising the paper and agreed to be accountable for all aspects of the work. All authors read and approved the final manuscript.

## Supporting information

Supporting Information

Supporting Information

## Data Availability

The data that support the findings of this study are available from the corresponding author upon reasonable request.
