# Supplementary material for: Overcoming Cancer Persister Cells by Stabilizing the ATF4 Promoter G‐quadruplex
Source: Adv Sci (Weinh). 2024 Jul 12;11(35):2401748. doi: 10.1002/advs.202401748 (PMC11425212; doi:10.1002/advs.202401748)
Supplement: Supplementary file 1 — Supporting Information [file ADVS-11-2401748-s001.docx]

Supporting Information

**Overcoming Cancer Persister Cells by Stabilizing the *ATF4* Promoter G-quadruplex**

*Chengmei Xiao^1^, Yipu Li^1^, Yushuang Liu^1^, Ruifang Dong, Xiaoyu He, Qing Lin, Xin Zang, Kaibo Wang*, Yuanzheng Xia* & Lingyi Kong**

*Corresponding authors: E-mail: kbwang@cpu.edu.cn (Kai-Bo Wang), xiayz@cpu.edu.cn (Yuan-Zheng Xia), cpu_lykong@126.com (Ling-Yi Kong).

This PDF file includes:

Figures S1 to S10

Tables S1 to S13





**Figure S1.** Glutamine restriction inhibits the proliferation of cancer cells. (A) Schematic depiction of glutamine metabolism in cancer cells: Glutamine plays a multifaceted role in cancer by serving as a crucial molecule involved in generating cell building blocks such as proteins, lipids, and nucleic acids. Glutamine enters cells through the cell membrane via SLC1A5 and is converted to glutamate by GLS (with GS catalyzing the reverse reaction). Glutamate is further metabolized to αKG, a substrate in the TCA cycle that ultimately leads to ATP production. Additionally, glutamine activates mTORC1 by facilitating the influx of leucine into cells through the bidirectional transporter SLC7A5. This process provides essential nutrients for the proliferation of cancer cells, suggesting that restricting glutamine availability could potentially inhibit tumor growth. To investigate the efficacy of glutamine-restricted therapies on lung cancer cells, we conducted a screening of various lung cancer cell lines to assess their sensitivity to glutamine restriction. GLS, glutaminase isoenzyme; GS, glutamine synthetase; TCA, tricarboxylic acid cycle; ROS, reactive oxygen species; αKG, α-ketoglutarate. (B) Survival rates of NCI-H460 and NCI-H1299 cells under varying concentrations of glutamine (4 mM, 0.5 mM, 0.25 mM, and 0 mM) for 48 h or 72 h (n = 3 independent experiments). (C) Trypan blue staining-based cell counting of NCI-H460 and NCI-H1299 cells treated with different glutamine concentrations for 1-5 days (n = 3 independent experiments). (D) EdU incorporation analysis in NCI-H460 and NCI-H1299 cells under different glutamine concentrations for 24 h. Scale bar: 200 μm. (E) Detection of ROS levels using flow cytometry with DCFH-DA dye in NCI-H460 and NCI-H1299 cells incubated with different glutamine concentrations for 12 h (n = 3 independent experiments). (F) NCI-H460 or NCI-H1299 cells were treated with 4 mM or 0.25 mM glutamine for 1-5 days, then cultured in complete medium for 2 h to generate persister cells, and the living cells were sorted and re-seeded into plate. Clonogenic survival of cells treated with glutamine, and 2 μM or 4 μM cisplatin (n = 3 independent experiments). Glutamine concentrations of 4 mM to 0 mM are represented by 4Q to 0Q, respectively. Data shown as mean ± SD. **p <* 0.05, ***p <* 0.01, ****p <* 0.001, *****p <* 0.0001. Data analyzed using two-tailed Student’s t-tests (C) and One-way ANOVA (B, E, F) in GraphPad Prism 9.5.0.


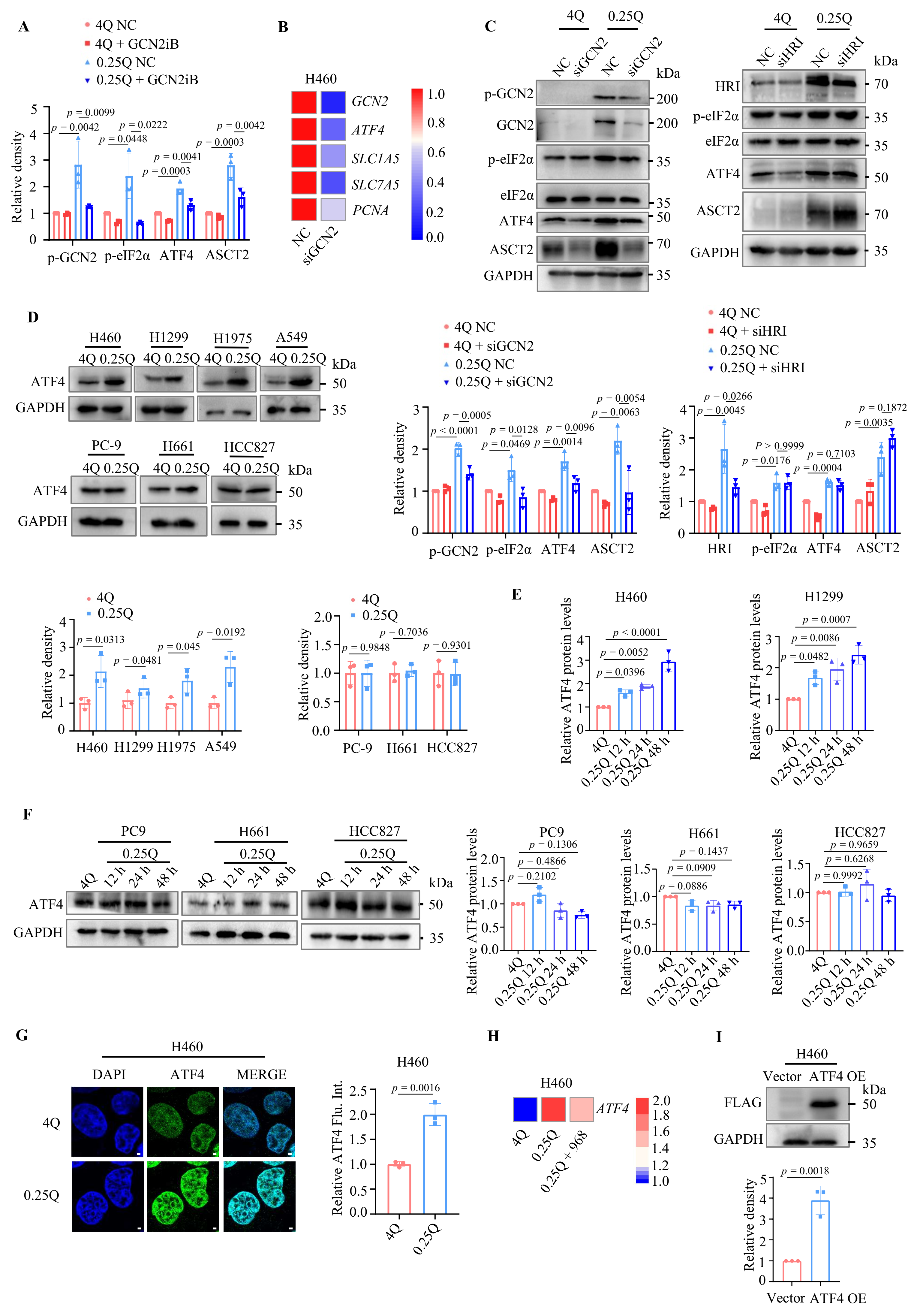


**Figure S2.** ISR induced by glutamine restriction promotes cancer survival via the GCN2-ATF4-ASCT2 axis. (A) Quantitative analysis of protein expression in NCI-H460 cells treated with GCN2iB by immunoblotting (n = 3 independent experiments). (B) Effect of knocking down *GCN2* on *ATF4*, *SLC1A5*, *SLC7A5*, and *PCNA* mRNA levels (n = 3 independent experiments, average of three technical replicates). (C) Immunoblotting and quantitative analysis of indicated proteins in NCI-H460 cells 48 h after treatment with siGCN2 or siHRI under 4 mM or 0.25 mM glutamine (n = 3 independent experiments). (D) Immunoblotting and quantitative analysis of ATF4 48 h after treatment with 4 mM or 0.25 mM glutamine in NCI-H460, NCI-H1299, NCI-H1975, A549, PC-9, NCI-H661, and HCC827 cells (n = 3 independent experiments). (E) Quantitative analysis of ATF4 expression in NCI-H460 and NCI-H1299 cells treated with 0.25 mM glutamine for 12 h, 24 h, and 48 h (n = 3 independent experiments). F) Immunoblotting and quantitative analysis of ATF4 expression in PC-9, NCI-H661, and HCC827 cells treated with 0.25 mM glutamine for 12 h, 24 h, and 48 h (n = 3 independent experiments). G) Immunofluorescence images and quantification of ATF4 expression in NCI-H460 cells treated with 4 mM or 0.25 mM glutamine. Scale bar: 10 μm. (n = 3 independent experiments). (H) qRT-PCR analysis of *ATF4* mRNA levels in NCI-H460 cells for 24 h after treatment with 968 (10 μM) under 0.25 mM glutamine (n = 3 independent experiments, average of three technical replicates). (I) Efficiency of overexpression of ATF4 in H460 cells (n = 3 independent experiments). OE: Overexpression ATF4, 4Q: 4 mM glutamine, 0.25Q: 0.25 mM glutamine. All immunoblots are representative of three biological replicates that showed similar results. Data shown as mean ± SD. **p <* 0.05, ** *p <* 0.01, ****p <* 0.001, *****p <* 0.0001. Data were analyzed using two-tailed Student’s t-tests (D, G, I) and One-way ANOVA (A, C, E, F) in GraphPad Prism 9.5.0.


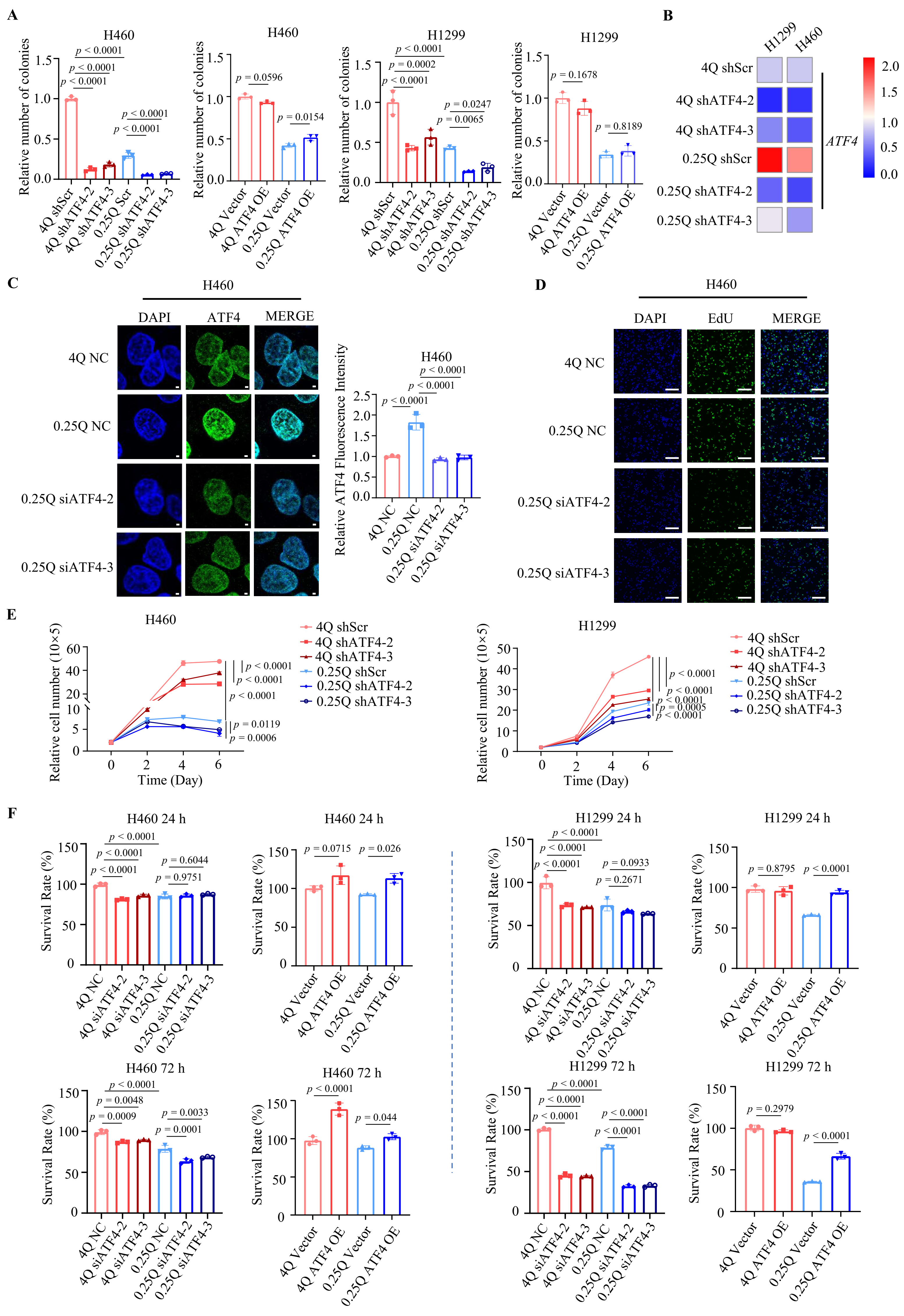


**Figure S3.** ATF4 is a key factor in glutamine restriction-induced ISR. (A) Quantitative analysis of clonogenic survival in NCI-H460 and NCI-H1299 cells transfected with shScramble, shATF4, Vector, and ATF4 OE plasmids under 4 mM or 0.25 mM glutamine (n = 3 independent experiments). (B) Knockdown efficiency of two independent *ATF4* shRNAs in NCI-H460 or NCI-H1299 cells (n = 3 independent experiments, average of three technical replicates). (C) Knockdown efficiency of two independent *ATF4* siRNAs in NCI-H460 cells analyzed by immunofluorescence. Scale bar: 10 μm (n = 3 independent experiments). (D) EdU incorporation analysis of 4 mM or 0.25 mM glutamine NC and 0.25 mM glutamine siATF4 in NCI-H460 cells for 48 h. Scale bar: 200 μm. (E) Cell counting of NCI-H460 or NCI-H1299 cells transfected with shScramble, shATF4 under 4 mM or 0.25 mM glutamine for 0, 2, 4, 6 days using trypan blue staining (n = 3 independent experiments). (F) Survival rate of NCI-H460 or NCI-H1299 cells transfected with NC, siATF4, Vector, and ATF4 OE plasmids under 4 mM or 0.25 mM glutamine for 24 h or 72 h (n = 3 independent experiments). OE: Overexpression ATF4, 4Q: 4 mM glutamine, 0.25Q: 0.25 mM glutamine. Data shown as mean ± SD. **p <* 0.05, ***p <* 0.01, ****p <* 0.001, *****p <* 0.0001. Data analyzed by One-way ANOVA (A, C, E, F) in GraphPad Prism 9.5.0.


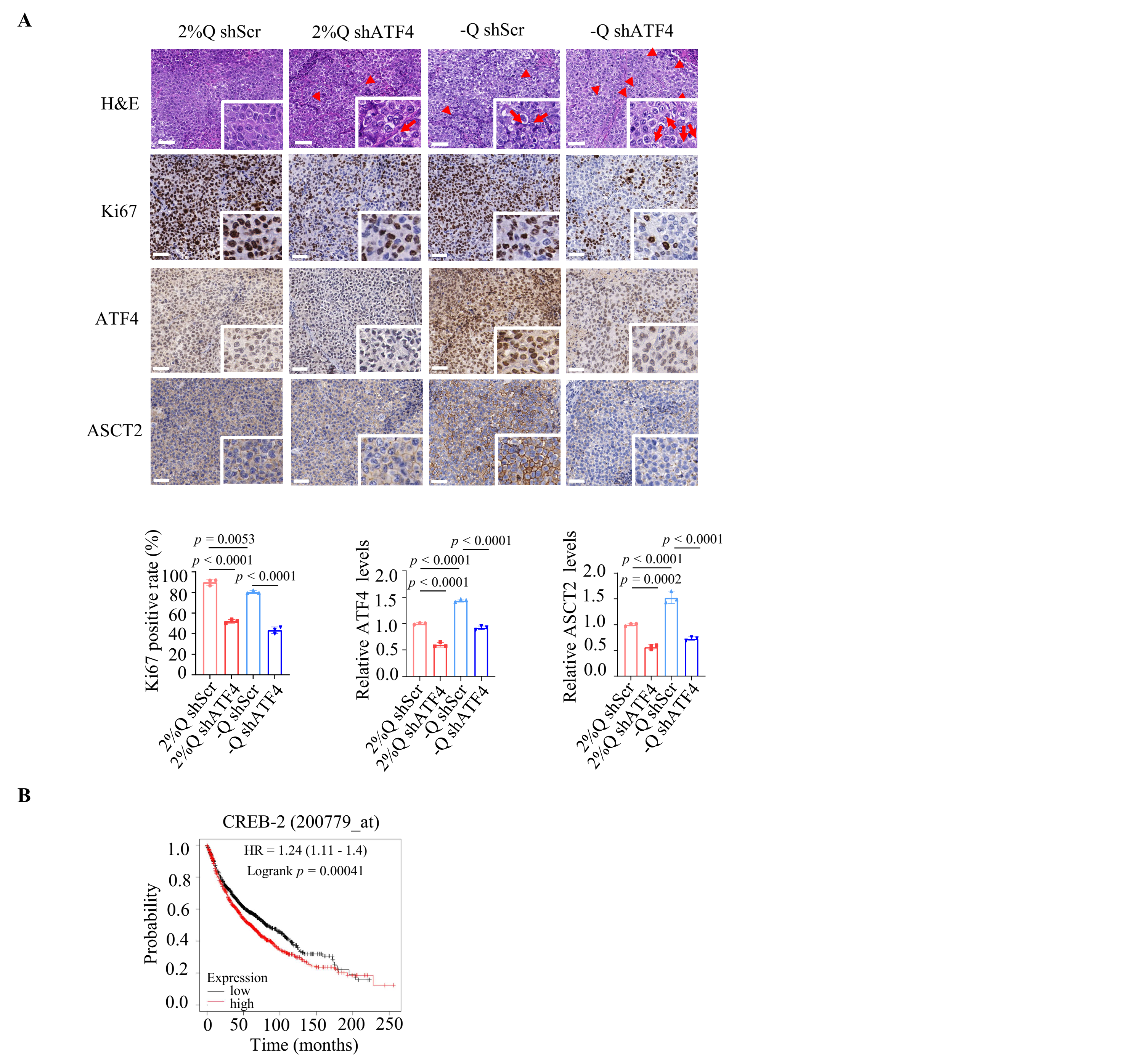


**Figure S4.** Low levels of ATF4 *in vivo* enhances sensitivity to glutamine restriction. (A) Representative immunohistochemical images of H&E, Ki67, ATF4, and ASCT2 with quantification shown (n = 3). Scale bar: 20 μm. (B) Kaplan-Meier survival analysis of ATF4. Data are shown as mean ± SD. ***p <* 0.01, ****p <* 0.001, *****p <* 0.0001. Data were analyzed using One-way ANOVA (A) in GraphPad Prism 9.5.0.


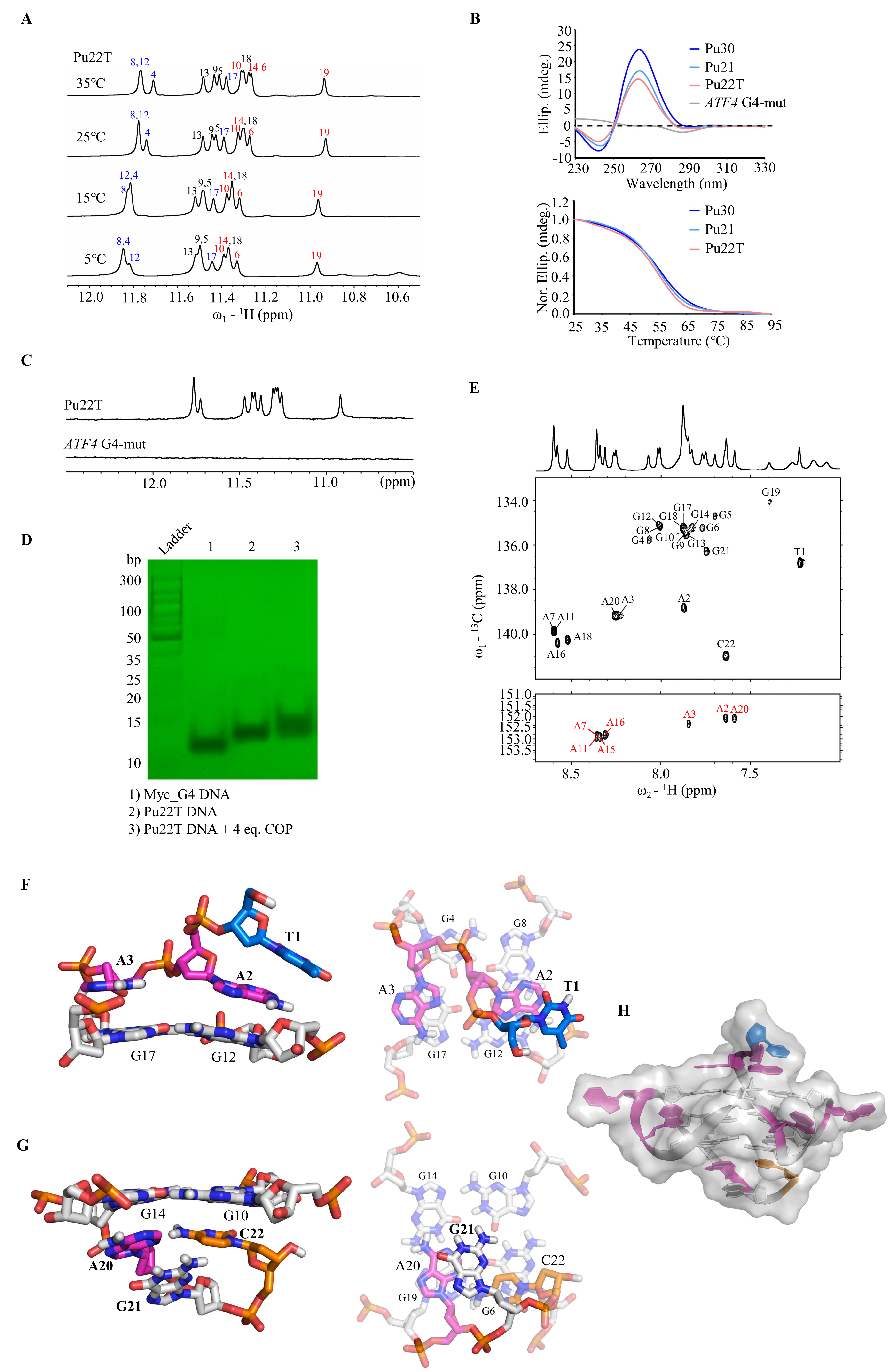


**Figure S5.** G-rich tracts presented in ATF4 promoter form a specific G-quadruplex. (A) 1D ^1^H-NMR spectra of *ATF4*-G4 with assignment at different temperatures. The G-tetrad imino proton signals at the 5′-end, middle, and 3′-end are labeled in blue, black, and red respectively. Conditions: 2.11 mM DNA, pH 7, 10 mM K^+^. (B) CD spectra and CD thermal melting curves of wild-type and mutant *ATF4* gene promoter sequences. Conditions: 20 μM DNA, pH 7, 5 mM K^+^. (C) 1D ^1^H-NMR spectra of Pu22T (*ATF4*-G4) and *ATF4*-G4 core mutant sequences. Conditions: 150 μM DNA, pH 7, 50 mM K^+^.

(D) Native EMSA gel of Myc-G4, Pu22T, and Pu22T+4.eq coptisine (COP). Conditions: 150 μM DNA, pH 7, 5 mM K^+^. Each sample contained 5 μL of 150 μM DNA. DNA bands were visualized using UV light at 254 nm. (E) H6-C6/H8-C8 cross-peaks for all bases (black label) and H2-C2 contacts for adenines (red label) with Pu22T DNA by ^1^H-^13^C HSQC experiments. Conditions: 1.65 mM Pu22T DNA, pH 7, 50mM K^+^-containing solution, 25 °C. (F, G) 5′-end, 3′-end side views and top views of *ATF4*-G4. (H) Surface superimposed on cartoon show the overall structure of *ATF4*-G4.


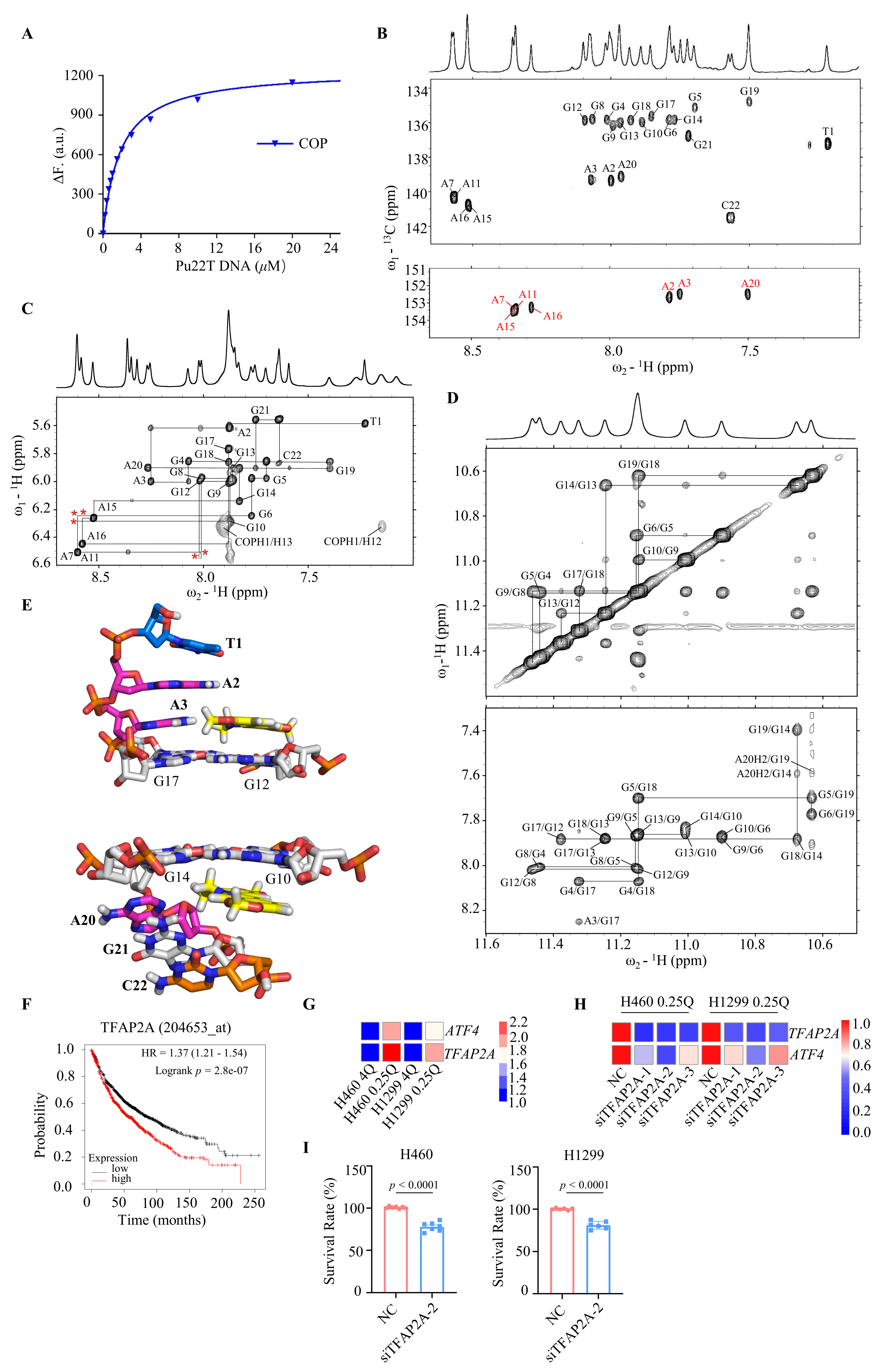


**Figure S6.** COP strongly binds and stabilizes the ATF4 promoter G4, the determination of COP-*ATF4*-G4 complex was realized. (A) Fluorescence intensity change of COP upon titration with Pu22T DNA. Conditions: 0.2 μM coptisine, pH 7, 50 mM K^+^ solution. (B) H6-C6/H8-C8 cross-peaks for all bases (black label) and H2-C2 contacts for adenines (red label) with assignments of COP-*ATF4*-G4 by ^1^H-^13^C HSQC experiments. Conditions: 2.11 mM Pu22T DNA, pH 7, 10 mM K^+^-containing solution, 25 °C. (C, D) H1′-H6/H8 region, H1-H1 and H1-H8 regions from the 2D-NOESY spectra of COP-Pu22T complex in H_2_O with sequential assignment pathway. Missing connectivity is marked with red asterisks. (E) 5′-end and 3′-end side views of COP-*ATF4*-G4. (F) Kaplan-Meier survival analysis of TFAP2A. (G) qRT-PCR analysis of ATF4 and TFAP2A mRNA expression levels in NCI-H460 or NCI-H1299 cells after 24 h treatment with 4 mM glutamine or 0.25 mM glutamine (n = 3 independent experiments, average of three technical replicates). Heatmap colors represent 2^-ΔΔCt^ values. (H) qRT-PCR analysis of the effect of *TFAP2A* knockdown on *ATF4* mRNA expression level (n = 3 independent experiments, average of three technical replicates). Heatmap colors represent 2^-ΔΔCt^ values. (I) Survival rate of NCI-H460 and NCI-H1299 cells transfected with siTFAP2A under 0.25 mM glutamine for 48 h (n = 3 independent experiments). 4Q: 4 mM glutamine, 0.25Q: 0.25 mM glutamine, COP: coptisine chloride. Data presented as mean ± SD. ****p <* 0.001. Data analyzed by two-tailed Student’s t-tests (I) in GraphPad Prism 9.5.0.


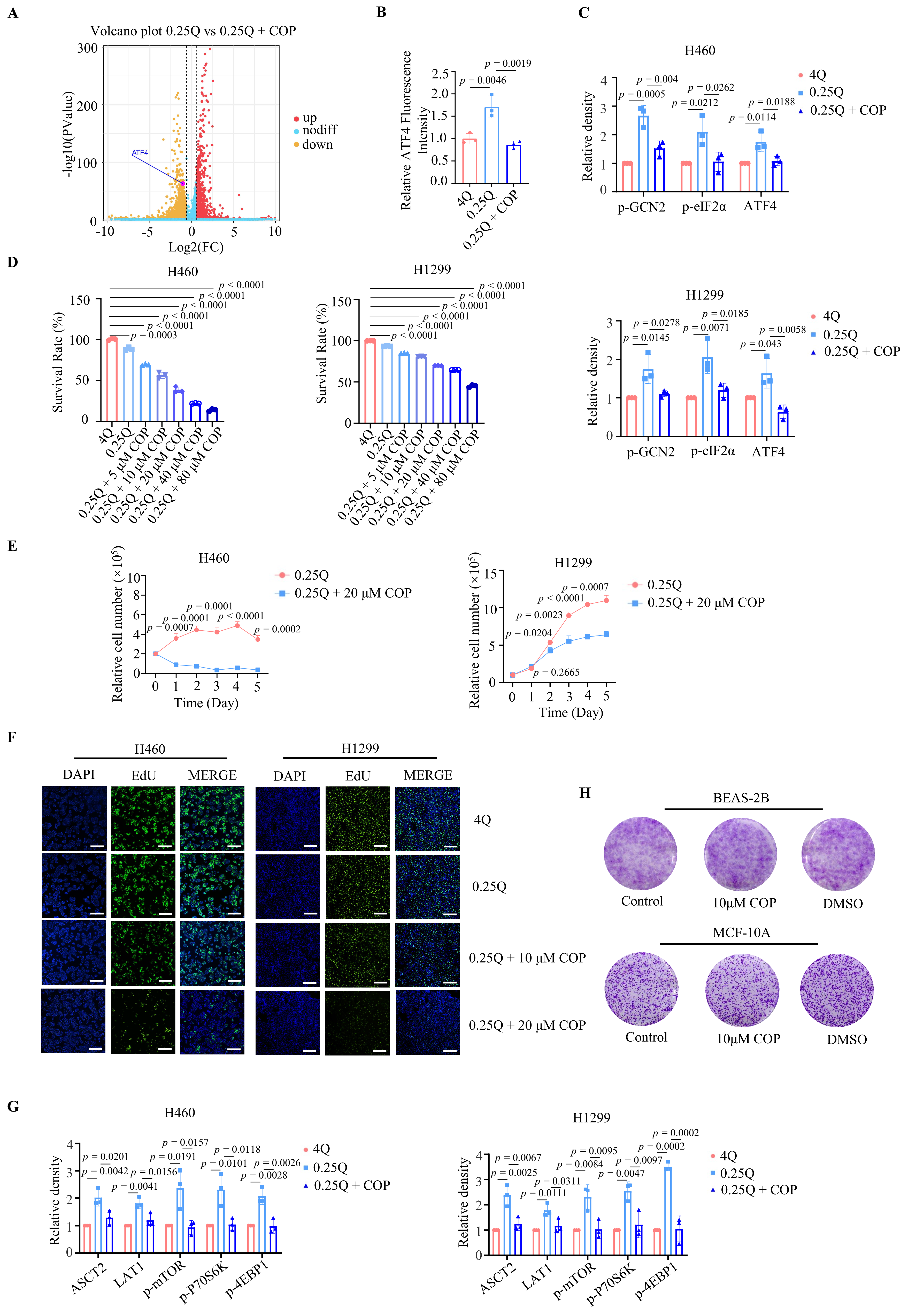


**Figure S7.** COP targets ATF4 to inhibit GCN2-ASCT2 axis thereby suppressing the proliferation of cancer cells. (A) RNA-Seq analysis compared differentially expressed genes in the H460 cell line (0.25Q vs 0.25Q + COP). The results showed a reduction in ATF4 gene expression, indicating that COP targets ATF4 to modulate glutamine metabolism. (n = 3 independent experiments). (B) Immunofluorescence quantification of the effect of 0.25 mM glutamine with COP (10 μΜ) (n = 3 independent experiments). (C) Immunoblotting and quantitative analysis of indicated proteins in NCI-H460 or NCI-H1299 cells after treatment with COP (20 μΜ) for 12 h under 0.25 mM glutamine (n = 3 independent experiments). (D) Survival rate of NCI-H460 and NCI-H1299 cells treated with varying concentrations of COP (5 μM, 10 μM, 20 μM, 40 μM, or 80 μM) under 4 mM or 0.25 mM glutamine for 48 h (n = 3 independent experiments). (E) Cell viability of NCI-H460 or NCI-H1299 cells treated with COP (20 μM) under 0.25 mM glutamine for 1-5 days, assessed by trypan blue staining (n = 3 independent experiments). (F) EdU staining in NCI-H460 or NCI-H1299 cells treated with 4 mM or 0.25 mM glutamine in the absence or presence of COP (10 μM or 20 μM) for 24 h. Scale bar: 200 μm. (G) Immunoblotting and quantitative analysis of indicated proteins in NCI-H460 or NCI-H1299 cells 48 h after treatment with COP (20 μΜ) under 0.25 mM glutamine (n = 3 independent experiments). (H) BEAS-2B and MCF-10A cells were used to assess the effect of COP (10 μM) on normal cells (n = 3 independent experiments). 4Q: 4 mM glutamine, 0.25Q: 0.25 mM glutamine, COP: coptisine chloride. Data are shown as mean ± SD. **p <* 0.05, ***p <* 0.01, ****p <* 0.001, *****p <* 0.0001. Data were analyzed by two-tailed Student’s t-tests (E) and One-way ANOVA (B-D, G) in GraphPad Prism 9.5.0.


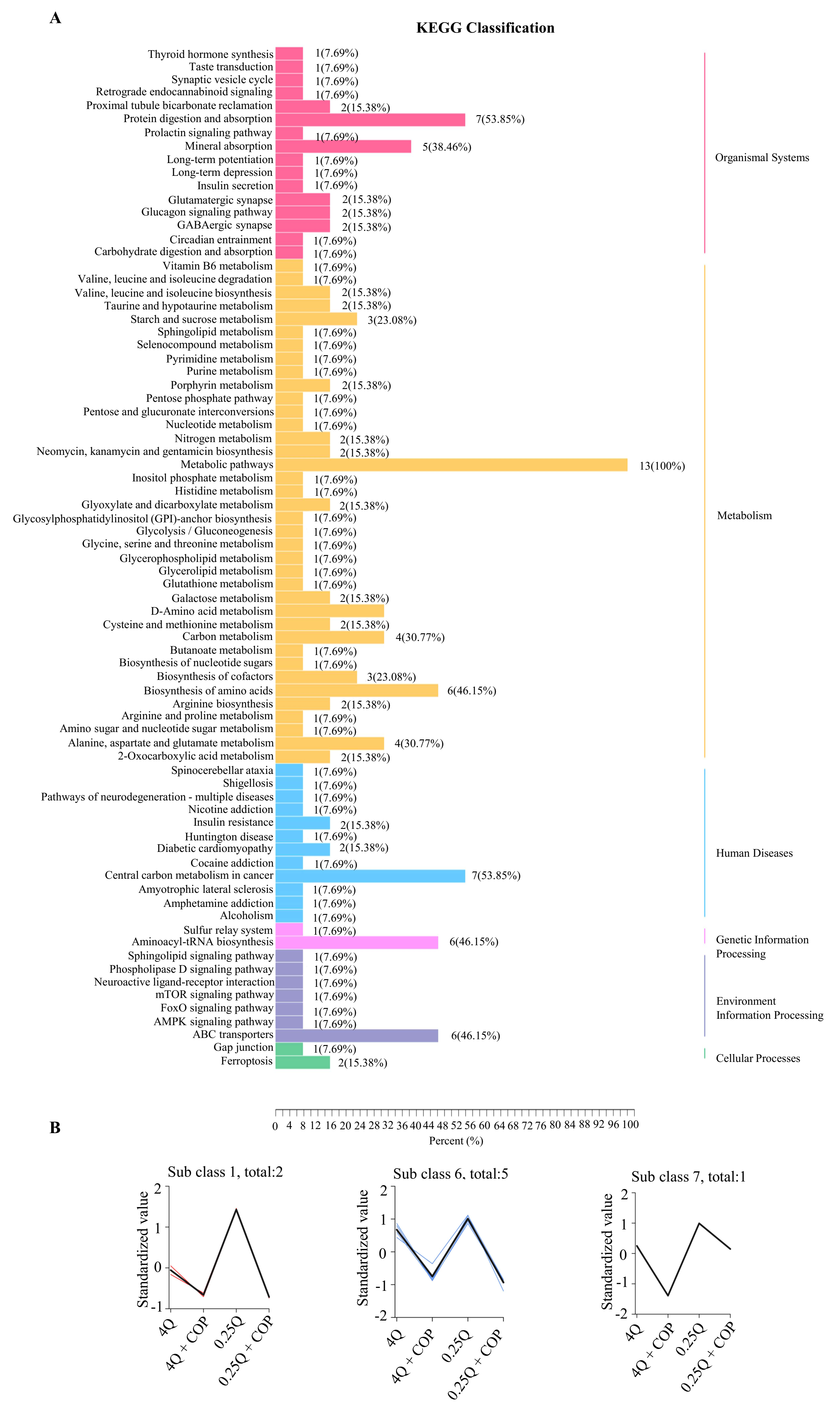


**Figure S8.** Metabonomic characteristics in NSCLC with COP and/or glutamine restriction treatment. (A) Classification of significantly different metabolites annotated to KEGG pathways in 0.25 mM glutamine and 0.25 mM glutamine with COP (20 μΜ) groups. The x-axis represents the proportion of annotated differential metabolites to the total annotated metabolites for each pathway, and the y-axis shows the names of KEGG metabolic pathways. (B) Metabolites of subgroups 1, 6, and 7: subgroup 1 includes threonine and L-asparagine; Subgroup 6 comprises glutamine, leucine, alanine, itaconic acid, and 6-phosphogluconic acid; Subgroup 7 contains 7-phosphate Sedum heptanose. The x-axis represents sample names, the y-axis indicates standardized relative content of metabolites, and Sub Class indicates metabolite category number with the same change trend. 4Q: 4 mM glutamine, 0.25Q: 0.25 mM glutamine, COP: coptisine chloride. Data are shown as mean ± SD.


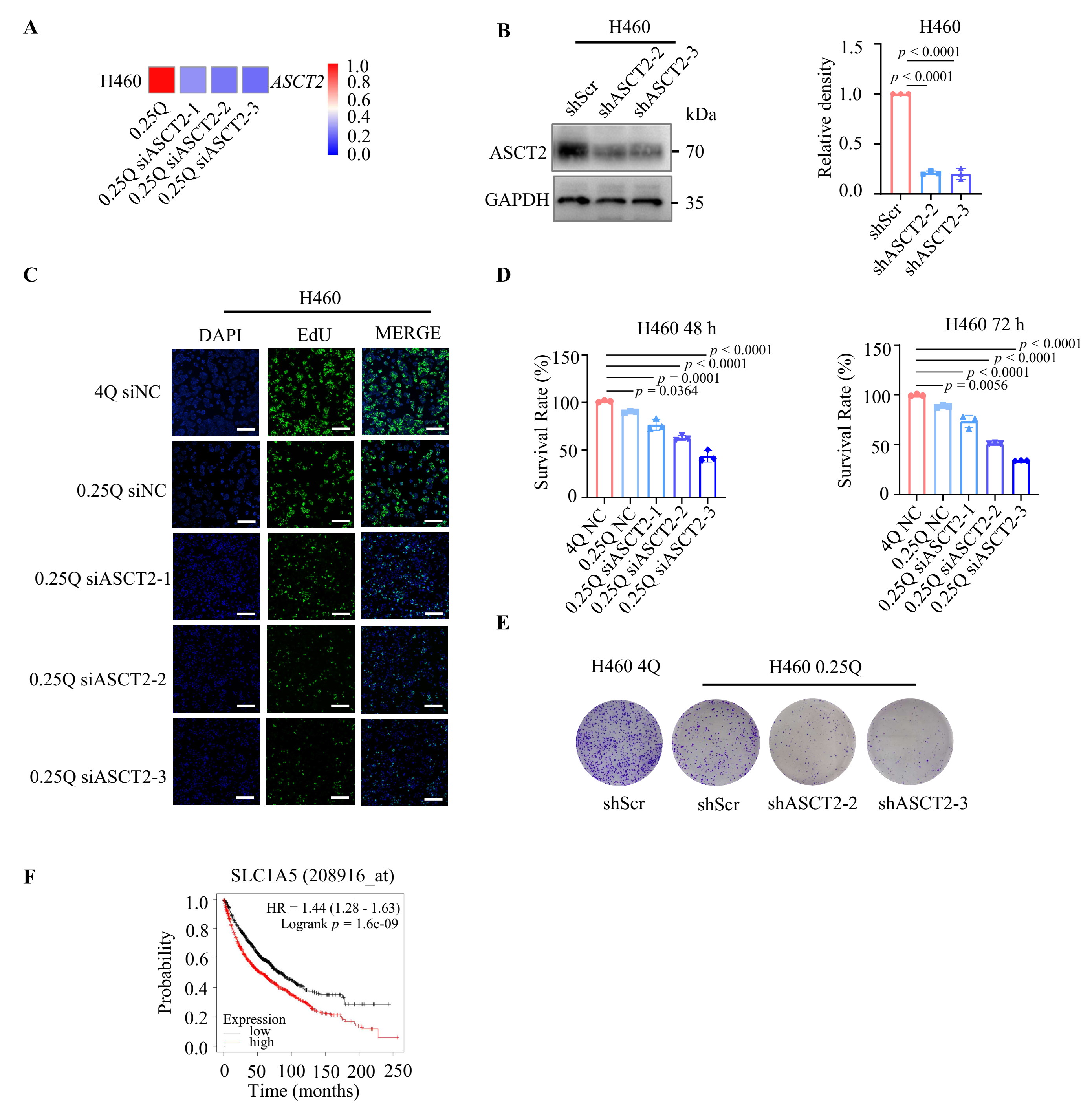


**Figure S9.** ASCT2 knockdown inhibits the proliferation of cancer cells. (A) Efficiency of ASCT2 knockdown by three independent ASCT2 siRNAs in NCI-H460 cells for 48 h (n = 3 independent experiments, average of three technical replicates). (B) Efficiency of ASCT2 knockdown by two independent shRNAs in NCI-H460 cells, assessed by Immunoblotting under 0.25 mM glutamine for 48 h (n = 3 independent experiments). (C) EdU incorporation assay of NCI-H460 cells treated with siASCT2 and 0.25 mM glutamine for 48 h. Scale bar: 200 μm. (D) Survival rate of NCI-H460 cells transfected with NC or siASCT2 under 0.25 mM glutamine for 48 h or 72 h (n = 3 independent experiments). (E) Clonogenic survival of NCI-H460 cells transfected with shScramble or shASCT2 under 0.25 mM glutamine. (F) Kaplan-Meier survival analysis of ASCT2. 4Q: 4 mM glutamine, 0.25Q: 0.25 mM glutamine. Data shown as mean ± SD. **p <* 0.05, ***p <* 0.01, ****p <* 0.001, *****p <* 0.0001. Data analyzed by One-way ANOVA (B, D) in GraphPad Prism 9.5.0.


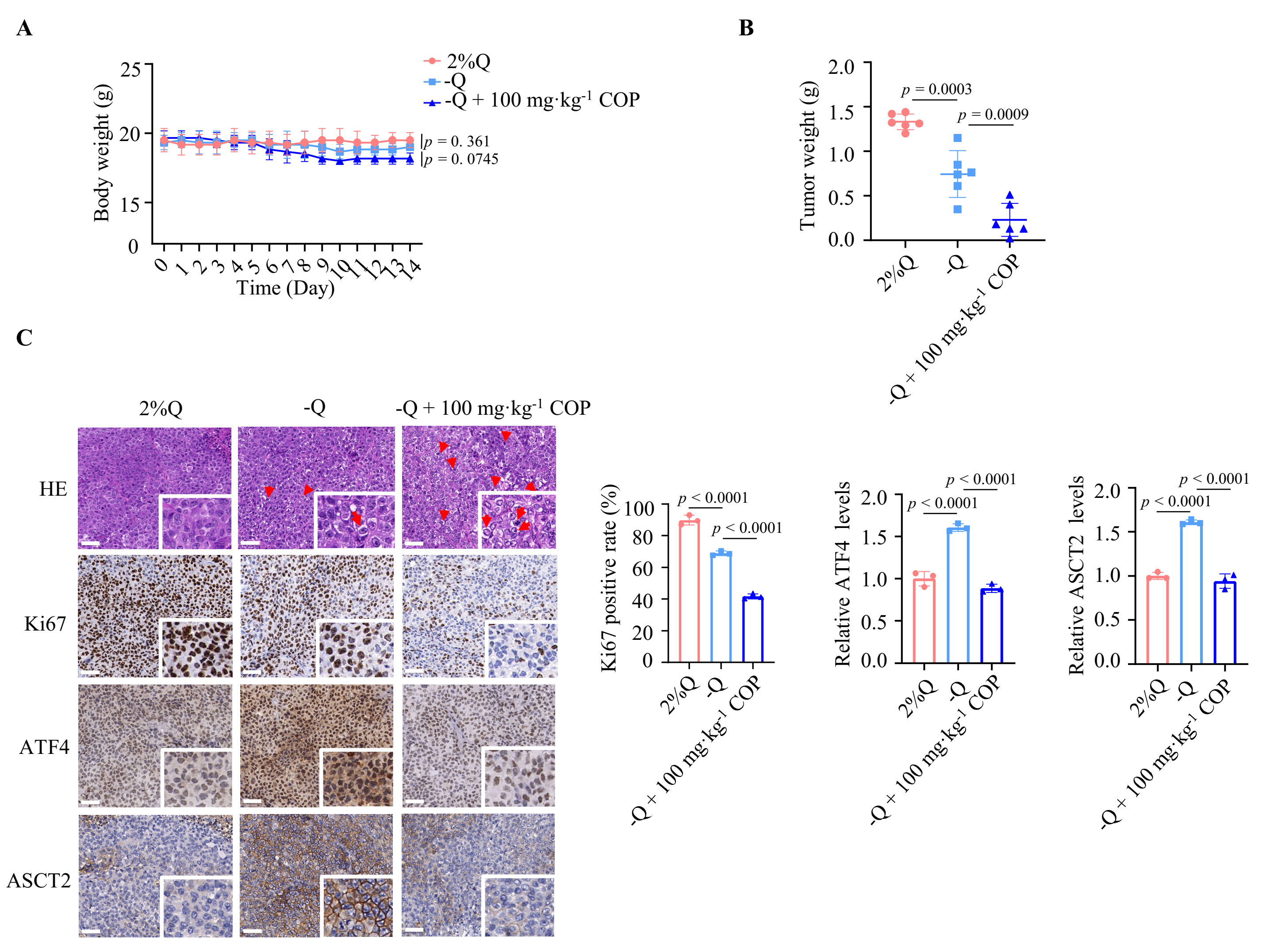


**Figure S10.** COP combined with glutamine restriction therapy enhances the anti-tumor effect. (A) Body weight of nude mice treated with COP (100 mg·kg^-1^) and glutamine restriction diet (n = 6 per group). (B) Tumor weight of nude mice treated with COP (100 mg·kg^-1^) and glutamine restriction diet (n = 6 per group). (C) Representative immunohistochemical images of H&E, Ki67, ATF4, and ASCT2 (left) and quantification (right) (n = 3). Scale bar: 20 μm. COP: coptisine chloride. Data are shown as mean ± SD. **p <* 0.05, ***p <* 0.01, ****p <* 0.001, *****p <* 0.0001. Data were analyzed by One-way ANOVA (A-C) in GraphPad Prism 9.5.0.

**Table S1. List of the primers used in qRT-PCR.**

| **Primer** | **Forward primer (5′ to 3′)** | **Reverse primer (5′ to 3′)** |
| --- | --- | --- |
| *TRIB3* | CAAGCTGTGTCGCTTTGTC | GAATCATCTGGCCCAGTCAG |
| *GPT2* | AGGAGTCCTTTGAGCAATTC | TTAAACAGGTCTTCCGTCAG |
| *MTHFD2* | CTCCTTGTTCAGTTGCCTCT | TCCTTGTCTGGAGAAACAGC |
| *PHGDH* | AGGAAGGGCATCTTGGTTAT | ATTCCACAAGTGAGTTCTGC |
| *PSAT1* | CAGAGAATCTTGTGCGGGAA | GTTTAAGGGGACAGCACTGA |
| *ADM2* | CGTCAAACCCAGGGAGC | GTGAAGCTTCCAGACCACAG |
| *ASNS* | CAGTTCGTGCTTCAGTAGGT | CGCTATCTGTGTTCTTCCGA |
| *SLC1A4* | GCAACTCAACAACGTAGAGC | TAATGGCAATGGTGAGGACC |
| *SLC1A5* | TCATGTGGTACGCCCCTGT | GCGGGCAAAGAGTAAACCCA |
| *SLC7A5* | CCGTGAACTGCTACAGCGT | CTTCCCGATCTGGACGAAGC |
| *SLC3A2* | CTGGTGCCGTGGTCATAATC | GCTCAGGTAATCGAGACGCC |
| *SLC7A1* | GCCTGTGCTATGGCGAGTTT | ACGCTTGAAGTACCGATGATGTA |
| *SLC7A11* | GCGTGGGCATGTCTCTGAC | GCTGGTAATGGACCAAAGACTTC |
| *SLC38A2* | ATGAGTTGCCTTTGGTGATCC | ACAGGACACGGAACCTGAAAT |
| *SLC38A1* | TGACAGTGCCCGAGGATGATA | AGACATGCCTAAGGAGGTTGTA |
| *GAPDH* | GGTGAAGGTCGGAGTCAACGG | GAGGTCAATGAAGGGGTCATTG |
| *ATF4* | ATGACCGAAATGAGCTTCCTG | GCTGGAGAACCCATGAGGT |
| *GLUL* | CCTGCTTGTATGCTGGAGTC | GATCTCCCATGCTGATTCCT |
| *E2F6* | TCCATGAACAGATCGTCATTGC | TCCGTTGGTGCTCCTTATGTG |
| *E2F4* | GGGCCTTTAGCCCTAGAACC | GAGGTGGGGCCAATCATCAG |
| *EWSR1* | GGATATGCACAGACCACCCA | GTCTGCCCATAGGTTGCAGT |
| *KLF1* | TTTCCCACGAATGGACCCTC | TTTGGCGGTCTGTCTCACTG |
| *USF1* | GCGGTCTTTCCGAGGCTTATC | GTCCCCTCTTCCGTTTCAGC |
| *NR2C2* | GGCGCCAAATCCTGAGGTAA | CTGTCCTGTCTGCTGGTCTG |
| *EGR1* | CCCCGACTACCTGTTTCCAC | TGGGTTTGATGAGCTGGGAC |
| *EGR2* | CAGGAGAGAGTCAGTGGCAAATA | CTCGCTACCTGGAGTGTCAGA |
| *TFAP2A* | TTGCTACACTGAGACTCCCG | CGTTCCTGGAGCCTCCTAAT |
| *SP1* | CCACCATGAGCGACCAAGAT | AAGGCACCACCACCATTACC |
| *EBF1* | CTTTCCAGCCCGCCTTGAT | ACTCCGTTGGATGCTTTCCT |
| *PAX5* | AAACCAAAGGTCGCCACAC | GTTGATGGAACTGACGCTAGG |
| *KLF4* | ACACACGGGATGATGCTCAC | ATCGTCTTCCCCTCTTTGGC |
| *ZEB1* | AGCTGTTTCAAGATGTTTCCTTC | CCTATGCTCCACTCCTTGCTAT |
| *FOXD3* | CTGCTTCGTCAAGATCCCCC | TAAGCGCCGAAGCTCTGCAT |
| *PDX1* | GATTGGCGTTGTTTGTGGCT | TTCCCCGCTGTGTGTGTTAG |
| *NKX3-2* | TAAGGAGGGGAGCCTCCAAG | TGCGACACGTCTTAACCTCC |
| *ZFX* | TTGCTGAAATCGCTGACGAAG | GCAATCGGCATGAAGGTTTTGAT |
| *MZF1* | TGCTGCCGTCTGTTGTACC | GGTGGTCGCAATGATCAGGT |
| *NFATC2* | GCACGCGGTAGAGAAGACG | TGCATTCGGCTCTTCTTGGT |
| *RUNX1* | AGGAAGTCAACCTCTGCTGC | CGGACCACAGAGCACTTTCT |
| *YY1* | GGATAACTCGGCCATGAGAA | ATAGGGCCTGTCTCCGGTAT |
| *PCNA* | CTGAGGGCTTCGACACCTAC | TCACTCCGTCTTTTGCACAG |
| *GCN2* | TGCCAACTTACATCAGAAAAGC | TTTGAGGTATATTTGCTTTGG |

**Table S2. List of the primers used in the ChIP.**

| **Primer** | **Forward primer (5′ to 3′)** | **Reverse primer (5′ to 3′)** |
| --- | --- | --- |
| *ATF4-*G4 | TGGGCATAAACGGTTGGGG | CTATGGGGACGCAGCACAGA |

**Table S3. List of the oligonucleotides used in the NMR, CD, Melting Curve Test, Kd Test, EMSA, DMS.**

| **Sequence Name** | **DNA Sequence (5′ to 3′)** |
| --- | --- |
| Pu21 | AAGGGAGGGAGGGAAGGGAGC |
| Pu22T  *ATF4* G4-mut | TAAGGGAGGGAGGGAAGGGAGC  TAAGAGAGAGAGAGAAGAGAGC |
| Pu30 | AAGGGAGGGAGGGAAGGGAGCGGGCGGGAG |
| 5′ Fam-Pu45 | CGTCAAAGGGAGGGAGGGAAGGGAGCGGGCGGGAGGAGACGGTCA |
| *Myc*-G4 | TGAGGGTGGGTAGGGTGGGTAA |

**Table S4.** **List of the antibodies used in the study.**

| **Primary Antibody** | **Company** | **Catalog number** | **Source** | **Dilution** |
| --- | --- | --- | --- | --- |
| ASCT2 | Proteintech | 20350-1-AP | Rabbit | WB: 1/2000, IHC: 1/400 |
| HRI | Proteintech | 20499-1-AP | Rabbit | WB: 1/1000 |
| TFAP2A | Proteintech | 13019-3-AP | Rabbit | WB: 1/2000 |
| p-P70S6K (Thr389) | Proteintech | 28735-1-AP | Rabbit | WB: 1/2000 |
| p70S6K | Proteintech | 14485-1-AP | Rabbit | WB: 1/2000 |
| SLC7A5 | Proteintech | 28670-1-AP | Rabbit | WB: 1/2000 |
| GLS | Proteintech | 23549-1-AP | Rabbit | IF : 1/200 |
| ATF4 | Abcam | ab184909 | Rabbit | WB: 1/500, IF : 1/200, IHC: 1/100 |
| mTOR | Abcam | ab134903 | Rabbit | WB: 1/10000 |
| p-mTOR (S2448) | Abcam | ab109268 | Rabbit | WB: 1/1000 |
| GCN2 | Abcam | ab134053 | Rabbit | WB: 1/1000 |
| eIF4EBP1 | Abcam | ab32024 | Rabbit | WB: 1/2000 |
| p-GCN2 (Thr899) | Affinity | AF8154 | Rabbit | WB: 1/1000 |
| GS | Affinity | DF7341 | Rabbit | IF : 1/200 |
| p-4EBP1 (Thr37/46) | Cell Signaling Technology | 2855 | Rabbit | WB: 1/1000 |
| eIF2α | Cell Signaling Technology | 5324S | Rabbit | WB: 1/1000 |
| p-eIF2α (Ser51) | Cell Signaling Technology | 3398 | Rabbit | WB: 1/1000 |
| Ki-67 | Cell Signaling Technology | 9449 | Mouse | IHC: 1/800 |
| GAPDH | Cell Signaling Technology | 97166 | Mouse | WB: 1/1000 |

**Table S5.** **Proton chemical shifts of *ATF4*-G4 at 15 °C in pH 7, 50 mM K^+^-containing solution.**

| **Base** | **H1/H2/H5** | **HMe** | **H6/H8** | **H1′** | **H2′, H2′′** | **H3′** | **H4′** | **H5′, H5′′*** |
| --- | --- | --- | --- | --- | --- | --- | --- | --- |
| **T1** |  | 1.71 | 7.24 | 5.72 | 1.58, 2.08 | 4.45 | 3.85 | 3.54, 3.54 |
| **A2** | 7.78 |  | 8.01 | 6.07 | 2.57, 2.65 | 4.82 | 4.20 | 3.75, 3.85 |
| **A3** | 7.76 |  | 8.11 | 5.92 | 2.50, 2.63 | 4.82 | 4.19 | 3.81, 3.82 |
| **G4** | 11.79 |  | 8.05 | 6.08 | 2.79, 3.01 | 5.03 | 4.51 | 4.11, 4.19 |
| **G5** | 11.46 |  | 7.73 | 6.23 | 2.65, 2.97 | 5.05 | 4.60 | 4.33, 4.33 |
| **G6** | 11.30 |  | 7.81 | 6.50 | 2.78, 2.65 | 5.18 | 4.70 | 4.37, 4.44 |
| **A7** | 8.35 |  | 8.58 | 6.73 | 2.97, 2.97 | 5.28 | 4.76 | 4.36, 4.36 |
| **G8** | 11.80 |  | 8.09 | 6.23 | 2.56, 3.03 | 5.23 | 4.56 | 4.35, 4.46 |
| **G9** | 11.47 |  | 8.02 | 6.29 | 2.73, 3.01 | 5.15 | 4.65 | 4.35, 4.35 |
| **G10** | 11.36 |  | 7.91 | 6.52 | 2.79, 2.62 | 5.18 | 4.72 | 4.39, 4.48 |
| **A11** | 8.35 |  | 8.58 | 6.73 | 2.97, 2.97 | 5.28 | 4.76 | 4.36, 4.36 |
| **G12** | 11.79 |  | 8.11 | 6.29 | 2.60, 3.03 | 5.26 | 4.58 | 4.36, 4.47 |
| **G13** | 11.50 |  | 7.99 | 6.17 | 2.73, 2.82 | 5.09 | 4.56 | 4.32, 4.36 |
| **G14** | 11.33 |  | 7.79 | 6.35 | 2.59, 2.53 | 4.98 | 4.28 | 3.73, 4.04 |
| **A15** | 8.35 |  | 8.53 | 6.45 | 2.86, 2.86 | 5.00 | 4.29 | 3.96, 4.00 |
| **A16** | 8.28 |  | 8.52 | 6.63 | 2.97, 2.96 | 5.19 | 4.63 | 4.29, 4.35 |
| **G17** | 11.41 |  | 7.87 | 5.95 | 2.38, 2.83 | 4.86 | 4.43 | 3.72，4.13 |
| **G18** | 11.33 |  | 7.96 | 6.06 | 2.78, 2.81 | 5.08 | 4.56 | 4.20, 4.28 |
| **G19** | 10.94 |  | 7.52 | 5.91 | 2.35, 2.64 | 5.00 | 4.50 | 4.20, 4.30 |
| **A20** | 7.49 |  | 7.99 | 5.81 | 2.38, 2.53 | 5.01 | 4.35 | 4.18, 4.18 |
| **G21**  **C22** | 5.68 |  | 7.74  7.56 | 5.69  6.01 | 2.45, 2.37  2.11, 2,25 | 4.82  4.42 | 4.10  3.98 | 4.03, 4.03  3.93, 3.93 |

**Table S6.** **Inter-residue NOEs of the *ATF4*-G4 5′-end capping structure.**

|  | **A2** |  |  |  |  |  | **A3** |
| --- | --- | --- | --- | --- | --- | --- | --- |
| **T1** | **H8** | **H2′** | **H2′′** | **H3′** | **H5′** | **H5′** | **H8** |
| **H6** | W |  |  |  |  |  |  |
| **H1′** | M | W | W | VW | VW | VW | W |
| **H2′** | W |  |  |  |  |  |  |
| **H2′′** | W |  |  |  |  |  |  |
| **H3′** | W | VW |  |  |  |  |  |
| **H4′** | OL | W | VW |  |  |  | W |
| **H5′** | VW |  |  |  |  |  |  |
| **H5′′** | VW |  |  |  |  |  |  |

|  | **A3** |  |  | **G4** | **G8** | **G12** | **G17** |
| --- | --- | --- | --- | --- | --- | --- | --- |
| **A2** | **H8** | **H5′** | **H5′′** | **H1** | **H1** | **H1** | **H1** |
| **H2** |  |  |  |  | W |  |  |
| **H8** | W |  |  | W | W | W | VW |
| **H1′** | W | W | W | W | W |  | VW |
| **H2′** | M |  |  | VW |  |  | VW |
| **H2′′** | M |  |  | W |  |  | W |
| **H3′** | M |  |  |  |  |  |  |
| **H4′** | OL |  |  |  |  |  |  |
| **H5′** | VW |  |  |  |  |  |  |
| **H5′′** | VW |  |  |  |  |  |  |

|  | **G4** |  |  |  | **G17** |  |
| --- | --- | --- | --- | --- | --- | --- |
| **A3** | **H1** | **H8** | **H5′** | **H5′′** | **H1** | **H1′** |
| **H2** |  |  |  |  | W | W |
| **H8** | OL |  |  |  | M |  |
| **H1′** |  | M | W | W | W |  |
| **H2′** |  | W |  |  |  |  |
| **H2′′** |  | W |  |  |  |  |
| **H3′** |  | W |  |  |  |  |
| **H4′** | VW | OL |  |  | W |  |
| **H5′** |  | W |  |  |  |  |
| **H5′′** |  | W |  |  |  |  |

Note: M = medium intensity, red marked, W = weak intensity, VW = very weak intensity, OL = overlapped cross-peak.

**Table S7.** **Inter-residue NOEs of the *ATF4*-G4 3′-end capping structure.**

|  | **G6** | **G14** | **G19** |  |  |  |  |  |  | **G21** |  |  |  |  | **C22** |
| --- | --- | --- | --- | --- | --- | --- | --- | --- | --- | --- | --- | --- | --- | --- | --- |
| **A20** | **H1** | **H1** | **H1** | **H8** | **H1′** | **H2′** | **H2′′** | **H3′** | **H4′** | **H8** | **H1′** | **H4′** | **H5′** | **H5′′** | **H5** |
| **H2** | VW | W | M |  |  |  |  |  |  |  | W |  |  |  | M |
| **H8** |  |  |  | M | W | M | M | W |  | W |  |  |  |  |  |
| **H1′** |  |  | VW |  |  |  |  |  |  | M |  | M | W | W |  |
| **H2′** |  |  |  |  |  |  |  |  |  | M |  |  |  |  |  |
| **H2′′** |  |  |  |  |  |  |  |  |  | S |  |  |  |  |  |
| **H3′** |  |  |  |  |  |  |  |  |  | W |  |  |  |  |  |
| **H4′** |  |  |  |  | W |  |  |  |  | W |  |  |  |  |  |
| **H5′** |  |  |  |  | OL |  |  |  |  |  |  |  |  |  |  |
| **H5′′** |  |  |  |  | OL |  |  |  |  |  |  |  |  |  |  |

|  | **C22** |  |
| --- | --- | --- |
| **G21** | **H5** | **H6** |
| **H1′** |  | M |
| **H2′** |  | W |
| **H2′′** |  | W |
| **H3′** |  | W |
| **H4′** | W | W |
| **H5′** |  | VW |
| **H5′′** |  | VW |

|  | **G6** |  | **G19** |
| --- | --- | --- | --- |
| **C22** | **H1** | **H1′** | **H1** |
| **H5** |  |  | **W** |
| **H6** | W |  |  |
| **H1′** | VW | W |  |
| **H2′** | W |  |  |
| **H2′′** | W |  |  |

Note: S = strong intensity, red marked, M = medium intensity, red marked, W = weak intensity, VW = very weak intensity, OL = overlapped cross-peak.

**Table S8.** **Inter-residue NOEs of the *ATF4*-G4 A15 and A16.**

|  | **G14** |  |  |  | **A16** |  |  |  |  |  |  | **G17** |  |  |  | **G18** |
| --- | --- | --- | --- | --- | --- | --- | --- | --- | --- | --- | --- | --- | --- | --- | --- | --- |
| **A15** | **H1′** | **H4′** | **H5′** | **H5′′** | **H8** | **H2′** | **H2′′** | **H3′** | **H4′** | **H5′** | **H5′′** | **H8** | **H2′** | **H2′′** | **H3′** | **H8** |
| **H2** | W | W | W | W |  |  |  |  |  |  |  |  | W | W | W |  |
| **H8** | M | W | W | W |  |  |  | VW | VW | W | W | W | W | OL | W | W |
| **H1′** |  |  |  |  | W | VW | VW | M | W | M | M |  |  |  |  |  |
| **H2′** |  |  |  |  | W |  |  |  |  |  |  |  |  |  |  |  |
| **H2′′** |  |  |  |  | W |  |  |  |  |  |  |  |  |  |  |  |
| **H3′** |  |  |  |  | W |  |  |  |  |  |  |  |  |  |  |  |
| **H4′** |  |  |  |  | OL |  |  |  |  |  |  |  |  |  |  |  |
| **H5′** |  |  |  |  | VW |  |  |  |  |  |  |  |  |  |  |  |
| **H5′′** |  |  |  |  | VW |  |  |  |  |  |  |  |  |  |  |  |

|  | **G17** |  |  |  |  |  |  |  |  |  |  |  |  |  |  |  |
| --- | --- | --- | --- | --- | --- | --- | --- | --- | --- | --- | --- | --- | --- | --- | --- | --- |
| **A16** | **H8** |  |  |  |  |  |  |  |  |  |  |  |  |  |  |  |
| **H2′** | VW |  |  |  |  |  |  |  |  |  |  |  |  |  |  |  |
| **H2′′** | VW |  |  |  |  |  |  |  |  |  |  |  |  |  |  |  |
| **H3′** | W |  |  |  |  |  |  |  |  |  |  |  |  |  |  |  |
| **H4′** | VW |  |  |  |  |  |  |  |  |  |  |  |  |  |  |  |

Note: M = medium intensity, red marked, W = weak intensity, VW = very weak intensity, OL = overlapped cross-peak.

**Table S9.** **Proton chemical shifts of Coptisine-*ATF4*-G4 at 25 °C in pH 7, 10 mM K^+^-containing solution.**

| **Base** | **H1/H2/H5** | **HMe** | **H6/H8** | **H1′** | **H2′, H2′′** | **H3′** | **H4′** | **H5′, H5′′*** |
| --- | --- | --- | --- | --- | --- | --- | --- | --- |
| **T1** |  | 1.69 | 7.23 | 5.79 | 1.56, 1.99 | 4.43 | 3.80 | 3.50, 3.50 |
| **A2** | 7.64 |  | 7.88 | 5.82 | 2.34, 2.36 | 4.76 | 4.43 | 4.07, 4.07 |
| **A3** | 7.85 |  | 8.25 | 6.20 | 2.68, 2.75 | 4.99 | 4.26 | 4.02, 4.06 |
| **G4** | 11.44 |  | 8.07 | 6.06 | 2.99, 2.74 | 5.01 | 4.51 | 4.18, 4.22 |
| **G5** | 11.15 |  | 7.70 | 6.18 | 2.63, 2.95 | 5.04 | 4.58 | 4.33, 4.33 |
| **G6** | 10.90 |  | 7.77 | 6.45 | 2.79, 2.66 | 5.18 | 4.67 | 4.34, 4.39 |
| **A7** | 8.36 |  | 8.60 | 6.71 | 2.96, 2.96 | 5.25 | 4.72 | 4.34, 4.34 |
| **G8** | 11.46 |  | 8.01 | 6.17 | 2.47, 2.98 | 5.18 | 4.54 | 4.32, 4.42 |
| **G9** | 11.15 |  | 7.87 | 6.20 | 2.65, 2.97 | 5.11 | 4.59 | 4.42, 4.44 |
| **G10** | 11.01 |  | 7.87 | 6.48 | 2.81, 2.64 | 5.18 | 4.68 | 4.34, 4.38 |
| **A11** | 8.36 |  | 8.60 | 6.71 | 2.96, 2.96 | 5.26 | 4.71 | 4.34, 4.34 |
| **G12** | 11.38 |  | 8.02 | 6.20 | 2.50, 2.95 | 5.19 | 4.57 | 4.34, 4.43 |
| **G13** | 11.25 |  | 7.86 | 6.10 | 2.67, 2.81 | 5.06 | 4.49 | 4.27, 4.32 |
| **G14** | 10.68 |  | 7.83 | 6.34 | 2.69, 2.59 | 5.03 | 4.32 | 3.83, 4.12 |
| **A15** | 8.34 |  | 8.53 | 6.46 | 2.86, 2.86 | 5.00 | 4.27 | 3.99, 3.99 |
| **A16** | 8.32 |  | 8.58 | 6.65 | 3.02, 2.95 | 5.20 | 4.63 | 4.33, 4.33 |
| **G17** | 11.32 |  | 7.88 | 5.97 | 2.41, 2.84 | 4.88 | 4.46 | 3.81, 4.13 |
| **G18** | 11.15 |  | 7.88 | 6.06 | 2.75, 2.75 | 5.09 | 4.55 | 4.22, 4.30 |
| **G19** | 10.63 |  | 7.40 | 6.11 | 2.12, 2.32 | 5.03 | 4.47 | 4.23, 4.30 |
| **A20** | 7.59 |  | 8.27 | 6.10 | 2.66, 2.68 | 4.99 | 4.44 | 4.08, 4.17 |
| **G21** |  |  | 7.75 | 5.76 | 2.51, 2.42 | 4.84 | 4.17 | 4.07, 4.07 |
| **C22** | 5.76 |  | 7..64 | 6.07 | 2.11, 2.24 | 4.84 | 4.43 | 3.99, 3.99 |

Note: *Assignments are not stereospecific.

**Table S10.** **Intermolecular NOEs between Coptisine and *ATF4*-G4.**

| 5′-end Coptisine | | | | | | | | | | |
| --- | --- | --- | --- | --- | --- | --- | --- | --- | --- | --- |
|  | **A2** | **A3** | **G4** | **G8** |  |  | **G12** |  | **G17** |  |
| **Coptisine** | **H2** | **H2** | **H1** | **H1** | **H1′** | **H8** | **H1** | **H8** | **H1** | **H8** |
| **H5** | W |  | W | W |  |  | W |  | W |  |
| **H6** | W |  | M | M |  |  | M |  | M |  |
| **H8** |  | W | W | W |  |  | W |  | W |  |
| **H12** |  |  |  |  |  |  |  | W |  |  |
| **HA** |  |  |  |  | M | W |  |  |  |  |
| **HB** |  |  |  |  |  |  |  |  |  | M |

| 3′-end Coptisine | | | | | | | | | | |
| --- | --- | --- | --- | --- | --- | --- | --- | --- | --- | --- |
|  | **A20** | **G6** |  |  | **G10** |  | **G14** |  | **G19** | **C22** |
| **Coptisine** | **H2** | **H1** | **H1′** | **H8** | **H1** | **H1′** | **H1** | **H8** | **H1** | **H4′** |
| **H5** | M | W |  |  | W |  | W |  | W |  |
| **H6** | W | M |  |  | M |  | M |  | M |  |
| **H8** |  | W |  |  | W |  | W |  | W |  |
| **H12** |  |  |  |  |  | W |  |  |  | W |
| **HA** |  |  | W | W |  |  |  |  |  |  |
| **HB** |  |  |  |  |  | W |  | W |  |  |

Note: M = medium intensity, red marked, W = weak intensity, VW = very weak intensity, OL = overlapped cross-peak.

**Table S11.** **Inter-residue NOE cross-peaks of the 5′-end capping structure of Coptisine-*ATF4*-G4 complex.**

|  | **A2** |  |  |  |  |  |  |
| --- | --- | --- | --- | --- | --- | --- | --- |
| **T1** | **H2** | **H8** | **H4′** | **H5′** | **H5′′** |  |  |
| **H6** | W | W |  |  |  |  |  |
| **H1′** |  | W | W | W | W |  |  |
| **H2′** |  | M |  |  |  |  |  |
| **H2′′** |  | M |  |  |  |  |  |
| **H3′** |  | W |  |  |  |  |  |

|  | **A3** |  |  |  |  |  |  |
| --- | --- | --- | --- | --- | --- | --- | --- |
| **A2** | **H1′** | **H2** | **H8** | **H3′** | **H4′** | **H5′** | **H5′′** |
| **H8** |  |  | W |  |  |  |  |
| **H1′** | M | W | M | W | W | M | M |
| **H2′** | W |  | M |  |  |  |  |
| **H2′′** | W |  | M |  |  |  |  |
| **H3′** |  |  | W |  |  |  |  |
| **H4′** |  |  | W |  |  |  |  |

|  | **G4** |  |  |  | **G12** | **G17** |
| --- | --- | --- | --- | --- | --- | --- |
| **A3** | **H1** | **H8** | **H5′** | **H5′′** | **H1** | **H1** |
| **H2** | W |  |  |  | VW | W |
| **H8** |  | W |  |  |  |  |
| **H1′** |  | M | W | W |  |  |
| **H2′** |  | W |  |  |  |  |
| **H2′′** |  | M |  |  |  |  |
| **H3′** |  | W |  |  |  |  |
| **H4′** |  | W |  |  |  |  |

Note: M = medium intensity, red marked, W = weak intensity, VW = very weak intensity, OL = overlapped cross-peak.

**Table S12.** **Inter-residue NOE cross-peaks of the 3′-end capping structure of Coptisine-*ATF4*-G4 complex.**

|  | **G14** | **G19** |  |  |  |  |  |  | **G21** |  |  |  |  |
| --- | --- | --- | --- | --- | --- | --- | --- | --- | --- | --- | --- | --- | --- |
| **A20** | **H1** | **H1** | **H8** | **H1′** | **H2′** | **H2′′** | **H3′** | **H4′** | **H8** | **H1′** | **H4′** | **H5′** | **H5′′** |
| **H2** | W | W |  |  |  |  |  |  |  | VW |  |  |  |
| **H8** |  |  | W | M | W | M | W | OL | W |  |  |  |  |
| **H1′** |  |  |  |  |  |  |  |  | W |  | W | W | W |
| **H2′** |  |  |  |  |  |  |  |  | M |  |  |  |  |
| **H2′′** |  |  |  |  |  |  |  |  | M |  |  |  |  |
| **H3′** |  |  |  |  |  |  |  |  | W |  |  |  |  |
| **H4′** |  |  |  | W |  |  |  |  | W |  |  |  |  |
| **H5′** |  |  |  | M |  |  |  |  |  |  |  |  |  |
| **H5′′** |  |  |  | M |  |  |  |  |  |  |  |  |  |

|  | **C22** |  |  |  |
| --- | --- | --- | --- | --- |
| **G21** | **H5** | **H6** | **H5′** | **H5′′** |
| **H8** | OL | W |  |  |
| **H1′** |  | OL | W | W |
| **H2′** |  | M |  |  |
| **H2′′** |  | M |  |  |
| **H3′** |  | W |  |  |
| **H4′** |  | W |  |  |

Note: S = strong intensity, red marked, M = medium intensity, red marked, W = weak intensity, VW = very weak intensity, OL = overlapped cross-peak.

**Table S13.** **Inter-residue NOEs of the Coptisine-*ATF4*-G4 complex A15 and A16.**

|  | **G14** |  |  |  | **A16** |  |  |  |  |  |  | **G17** |  |  |  | **G18** |
| --- | --- | --- | --- | --- | --- | --- | --- | --- | --- | --- | --- | --- | --- | --- | --- | --- |
| **A15** | **H1′** | **H4′** | **H5′** | **H5′′** | **H8** | **H2′** | **H2′′** | **H3′** | **H4′** | **H5′** | **H5′′** | **H8** | **H2′** | **H2′′** | **H3′** | **H8** |
| **H2** | W | W | W | W |  |  |  |  |  |  |  |  | W | W | W |  |
| **H8** | M | W | W | W |  |  |  | VW | VW | W | W | W | W | OL | W | W |
| **H1′** |  |  |  |  | W | VW | VW | M | W | M | M |  |  |  | W |  |
| **H2′** |  |  |  |  | W |  |  |  |  |  |  |  |  |  |  |  |
| **H2′′** |  |  |  |  | W |  |  |  |  |  |  |  |  |  |  |  |
| **H3′** |  |  |  |  | W |  |  |  |  |  |  |  |  |  |  |  |
| **H4′** |  |  |  |  | OL |  |  |  |  |  |  |  |  |  |  |  |
| **H5′** |  |  |  |  | VW |  |  |  |  |  |  |  |  |  |  |  |
| **H5′′** |  |  |  |  | VW |  |  |  |  |  |  |  |  |  |  |  |

|  | **G17** |  |  |  |  |  |  |  |  |  |  |  |  |  |  |  |
| --- | --- | --- | --- | --- | --- | --- | --- | --- | --- | --- | --- | --- | --- | --- | --- | --- |
| **A16** | **H8** | **H3′** | **H5′** | **H5′′** |  |  |  |  |  |  |  |  |  |  |  |  |
| **H1′** |  | W | VW | VW |  |  |  |  |  |  |  |  |  |  |  |  |
| **H4′** | W |  |  |  |  |  |  |  |  |  |  |  |  |  |  |  |

Note: M = medium intensity, red marked, W = weak intensity, VW = very weak intensity, OL = overlapped cross-peak.
